# Proteomic Signatures of Monocytes in Hereditary Recurrent Fevers

**DOI:** 10.3389/fimmu.2022.921253

**Published:** 2022-06-23

**Authors:** Federica Penco, Andrea Petretto, Chiara Lavarello, Riccardo Papa, Arinna Bertoni, Alessia Omenetti, Ilaria Gueli, Martina Finetti, Roberta Caorsi, Stefano Volpi, Marco Gattorno

**Affiliations:** ^1^Centro Malattie Autoinfiammatorie ed Immunodeficienze, Istituto di Ricovero e Cura a Carattere Scientifico (IRCCS) Istituto Giannina Gaslini, Genova, Italy; ^2^Core Facilities - Clinical Proteomics and Metabolomics, Istituto di Ricovero e Cura a Carattere Scientifico (IRCCS) Istituto Giannina Gaslini, Genoa, Italy; ^3^Pediatric Unit, Department of Mother and Child Health, Salesi Children’s Hospital, Ancona, Italy; ^4^Clinica Pediatrica e Reumatologica, Istituto di Ricovero e Cura a Carattere Scientifico (IRCCS) Istituto Giannina Gaslini, Genova, Italy

**Keywords:** Hereditary recurrent fevers, inflammation, proteomic, monocytes, signature

## Abstract

Hereditary periodic recurrent fevers (HRF) are monogenic autoinflammatory associated to mutations of some genes, such as diseases caused by mutations of including MEFV, TNFRSF1A and MVK genes. Despite the identification of the causative genes, the intracellular implications related to each gene variant are still largely unknown. A large –scale proteomic analysis on monocytes of these patients is aimed to identify with an unbiased approach the mean proteins and molecular interaction networks involved in the pathogenesis of these conditions. Monocytes from HRF 15 patients (5 with MFV, 5 TNFRSF1A and 5with MVK gene mutation) and 15 healthy donors (HDs) were analyzed by liquid chromatography and tandem mass spectrometry before and after lipopolysaccharide (LPS) stimulation. Significant proteins were analyzed through a Cytoscape analysis using the ClueGo app to identify molecular interaction networks. Protein networks for each HRF were performed through a STRING database analysis integrated with a DISEAE database query. About 5000 proteins for each HRF were identified. LPS treatment maximizes differences between up-regulated proteins in monocytes of HRF patients and HDs, independently from the disease’s activity and ongoing treatments. Proteins significantly modulated in monocytes of the different HRF allowed creating a disease-specific proteomic signatures and interactive protein network. Proteomic analysis is able to dissect the different intracellular pathways involved in the inflammatory response of circulating monocytes in HRF patients. The present data may help to identify a “monocyte proteomic signature” for each condition and unravel new possible unexplored intracellular pathways possibly involved in their pathogenesis. These data will be also useful to identify possible differences and similarities between the different HRFs and some multifactorial recurrent fevers.

## Highlights

- Each HRF is characterized by a specific “monocyte proteomic signature” upon standard stimulation, that may act as a biomarker for its recognition
- Proteomics data from unstimulated and stimulated monocytes may highlight possible novel and unexplored intracellular pathways involved in HRFs pathogenesis.
- A comparison with non-monogenic multifactorial recurrent fevers (PFAPA, SURF) could help the identification of possible common pathways and therapeutic targets.

## Introduction

Hereditary periodic recurrent fevers (HRF) are monogenic autoinflammatory associated to mutations of some genes, such as diseases caused by mutations of including MEFV, TNFRSF1A and MVK genes (1–3). Familial Mediterranean fever (FMF) is the prototype of a HRF and is caused by gain-of-function mutations of the *MEFV* gene, coding for pyrin (4, 5). Pyrin is part of the Pyrin-Inflammasome that controls the activation and secretion of interleukin (IL)-1β and can be activated by the cortical actin cytoskeleton remodeling induced by bacterial toxins *via* RhoA. Mevalonate kinase deficiency (MKD) is secondary to bi-allelic mutations of the gene coding for mevalonate kinase (*MVK*), an enzyme involved in the cholesterol biosynthesis. Loss-of-function *MVK* mutations lead to a defective sterol synthesis and to a reduced prenylation of small G proteins, such as RhoA, favoring the activation of the Pyrin-inflammasome (6–8). TNF receptor-associated periodic syndrome (TRAPS) is secondary to autosomal dominant mutations of the gene coding for the type I receptor for TNF (*TNFRSF1A)*, resulting in an accumulation of misfolded TNF receptor 1 in the endoplasmic reticulum, leading to oxidative stress, defective autophagy and consequent over-secretion of pro-inflammatory cytokines (9, 10).

The clinical manifestations of HRFs are rather unspecific and consist mainly in fever, serositis, skin rash, limb and articular pain. No specific biomarkers able to distinguish one among the different HRFs have been identified (11). The majority of the studies investigating the pathogenic consequences of the genes’ mutations associated with the three HRFs were focused on the few mechanisms classically associated to the pro-inflammatory activation of innate immunity cells (NF-kB and MAPK activation, IL-1β activation and secretion). Conversely, an unbiased analysis of possible additional intracellular pathways involved in the different conditions has not been exploited. Recently, large-scale proteomics based on high-resolution mass spectrometry (MS) has offered a systems-wide hypothesis-free method to analyze intracellular pathways in order to identify new disease biomarkers (12–14). Here, we analyzed the proteomic signature of unstimulated and stimulated monocytes of patients with FMF, TRAPS and MKD, describing the dysregulated intracellular pathways associated with each condition to increase knowledge on their possible involvement the pathogenesis of these diseases.

## Methods

### Samples Collection

Blood samples from 15 patients with HRFs (5 TRAPS, 5 FMF, 5 MKD) and 15 age matched healthy donors (HD) were obtained during the routine follow-up, collected in EDTA-coated Vacutainer tubes and processed the same day of the collection. Clinicians evaluated the disease activity considering the presence of symptoms related to each HRF. Laboratory tests were considered abnormal if C-reactive protein (CRP) was > 0.46 mg/dl and serum amyloid A (SAA) was > 10 mg/l. Five consecutive patients were chosen with the following inclusion criteria: i) presence of a confirmatory genotype (exclusion of variant of unknown significance), satisfactory control of the disease and/or absence of a frank disease flare at the moment of sampling, iii) absence of relevant co-morbidities.

The study was approved by the Ethics Committee of the Gaslini Institute, all participants gave written informed consent.

### Monocytes Activation and Samples Preparation for MS Analysis

Peripheral blood mononuclear cells (PBMCs) were isolated by gradient centrifugation (Ficoll Hystpaque) and then purified with CD14 microbeads positive selection (Miltenyi Biotec). Then, monocytes were cultured (15, 16) for 4h in culture medium Rosewell Park Memorial Institute (RPMI) 1640 supplemented with 5% fetal calf serum (FCS) in the presence or absence of LPS (100 ng/ml; Sigma Aldrich). The treatment with lipopolysaccharide (LPS) was used to minimize the impact of the disease activity and/or on-going therapy and to investigate the difference in the responsiveness of mutated monocytes in respect to healthy controls. Monocytes were analyzed by high-resolution liquid chromatography and tandem MS (17). The cellular pellets were resuspended in 25 μl of the lysis buffer (6M GdmCl, 10mM TCEP, 40mM CAA, 100mMTris pH8.5). The samples were reduced, alkylated and lastly digested in a single step, and loaded into the Stage Tip (17). Peptides were analyzed by nano-UHPLC-MS/MS using an Ultimate 3000 RSLC with EASY spray column (75 μm x 500 mm, 2 μm particle size, Thermo Scientific) and with a 180-minute nonlinear gradient of 5-45% solution B (80% CAN and 20% H2O, 5% DMSO, 0.1% FA) at a flow rate of 250 nl/min. Eluting peptides were analyzed using an Orbitrap Fusion Tribrid mass spectrometer (Thermo Scientific Instruments, Bremen, Germany). Orbitrap detection was used for both MS1 and MS2 measurements at resolving powers of 120 K and 30 K (at m/z 200), respectively. Data-dependent MS/MS analysis was performed in top speed mode with a 3 seconds cycle time, during which precursors detected within the range of 375−1500 m/z were selected for activation in order of abundance. Quadrupole isolation with a 1.4 m/z isolation window was used and dynamic exclusion was enabled for 45 s. Automatic gain control targets were 2.5 × 10^5 for MS1 and 5 × 10^4 for MS2, with 50 and 45 ms maximum injection times, respectively. The signal intensity threshold for MS2 was 1 × 10^4. HCD was performed using 30% normalized collision energy. One Microscan was used for both MS1 and MS2 events.

### Statistical Analysis and Network Analysis

MaxQuant software was used to process the MS raw data, setting a false discovery rate (FDR) of 0.01 for the identification of proteins, peptides and peptide-spectrum matches. Moreover, a minimum length of six amino acids was required for peptide identification. The MaxQuant-incorporated Andromeda engine was used to search MS/MS spectra against the Uniprot human database. For protein digestion, allowing for N-terminal to proline cleavage, trypsin was chosen. Cysteine carbamidomethylation was selected as fixed modification, whereas N-terminal protein acetylation, oxidation (M) and deamidation (N, Q) have been selected as variable modifications. A tolerance of 7 ppm was set for the mass deviation of the precursor ions, while the maximum mass deviation for MS2 events was 0.5 Da. Algorithm MaxLFQ (12) was chosen for protein quantification, with the activated option ‘match between runs’ to reduce the number of missing proteins. All bioinformatics analyses were done with the Perseus software of the MaxQuant computational platform (13). Protein groups were filtered up to require 70% valid values in at least one experimental group. The label-free intensities were expressed as base log2, and empty values were imputed with random numbers from a normal distribution for each column, to best simulate the low abundance values close to the noise level. For each group, a t-test with permutation-based FDR of 0.05 and as 0 of 0.1 was used. The Venn diagram of identified proteins was calculated using an online tool (14). A Student’s t-test analysis (FDR < 0.05 and S0 > 0.1) was performed to characterize differences and reciprocal relationships between the two groups of each HRF. Principal Component Analysis (PCA) was performed to visualize similarities or differences between samples. The significant proteins were plotted using a Volcano plot. Cytoscape analysis by ClueGo app was performed to identify biological functions of t-test significantly modulated proteins, highlighting which pathways were altered in each disease. Similarly, using the STRING app on the significantly modulated proteins, the protein-protein interaction network has been constructed (18–20). The network was then annotated with the DISEASE database in order to highlight for each disorder the known and unknown proteins that correlate with HRF and its intracellular pathways. The mass spectrometry proteomics data have been deposited to the ProteomeXchange Consortium *via* the PRIDE (21) partner repository with the dataset identifier PXD024068.

## Results

### Proteins Quantification and Identification

The clinical characteristics of the enrolled patients are reported in **Table 1**. The patients were sampled between fever attacks. Three patients (2 FMF and 1 MKD) displayed a subclinical elevation of the acute phase reactants. One MKD patient had a flare in the few days before the sampling. 4 FMF patients were in treatment with Colchicine; all TRAPS patients and 3 MKD patients were in treatment with Canakinumab; the remaining patients were not on therapy at the time of collection.

**Table 1:** Characteristics of patients.

N°	Diagnosis	Mutation	Disease activity	CRP (mg/dL)	SAA (mg/L)	Treatment
1	FMF	M694V/M694V	Mild	1,82	145	Colchicine
2	FMF	M694V/V726A	Almost absent	1,16	26	Colchicine
3	FMF	M694V/V726A	None	<0,46	15,3	None
4	FMF	M680I/V726A	None	<0,46	14,2	Colchicine
5	FMF	M694V/M694V	None	<0,46	4,2	Colchicine
6	TRAPS	C55Y	None	<0,46	2,07	Canakinumab
7	TRAPS	C88Y	None	<0,46	1,8	Canakinumab
8	TRAPS	C52Y	None	<0,46	5,39	Canakinumab
9	TRAPS	T50M	None	<0,46	9,94	Canakinumab
10	TRAPS	C55Y	None	<0,46	3,86	Canakinumab
11	MKD	L265R/V377I	None	<0,46	4,1	Canakinumab
12	MKD	C605insG/V377I	Mild	1,21	135	Canakinumab
13	MKD	C785_790delC/V377I	None	<0,46	7,2	Canakinumab
14	MKD	V310M/V377I	Moderate	3,08	130	None
15	MKD	V377I/V377I	None	<0,46	16,8	None

FMF, familial Mediterranean fever; TRAPS, TNF receptor-associated periodic syndrome; MKD, mevalonate kinase deficiency; CPR, C-reactive protein; SAA, serum amyloid A.

**Figure 1:**
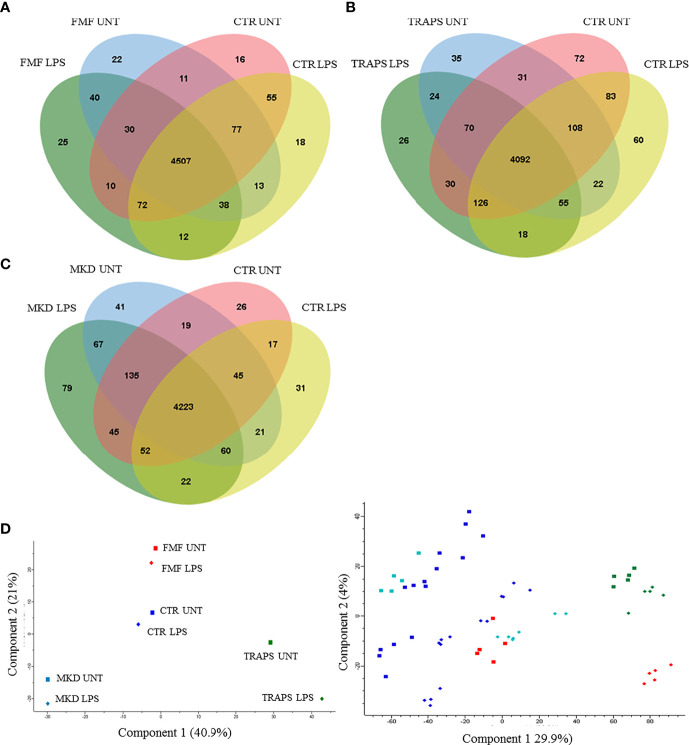
Venn diagram shows the number of proteins in untreated and LPS-treated monocytes of patients with FMF **(A)**, TRAPS **(B)**, and MKD **(C)** compared to HD. Principal component analysis **(D)** shows discrimination between proteins of untreated (squares) and LPS-treated (rhombus) monocytes of patients with FMF (red), TRAPS (green), MKD (light blue) and HD (blue). The left panel represents the components’ means distribution of the different samples for each group of HRF and HD; the right panel represents the distribution of the individual samples for each group of HRF and HD.

Monocytes of HRF patients and HDs with or without LPS stimulation were analyzed and quantified by MS. Almost 5000 proteins for each HRF were identified (**Figures 1A–C**), with an average of about 4000 proteins for groups, at a peptide and false discovery rate (FDR) of 1%.

**Figure 2:**
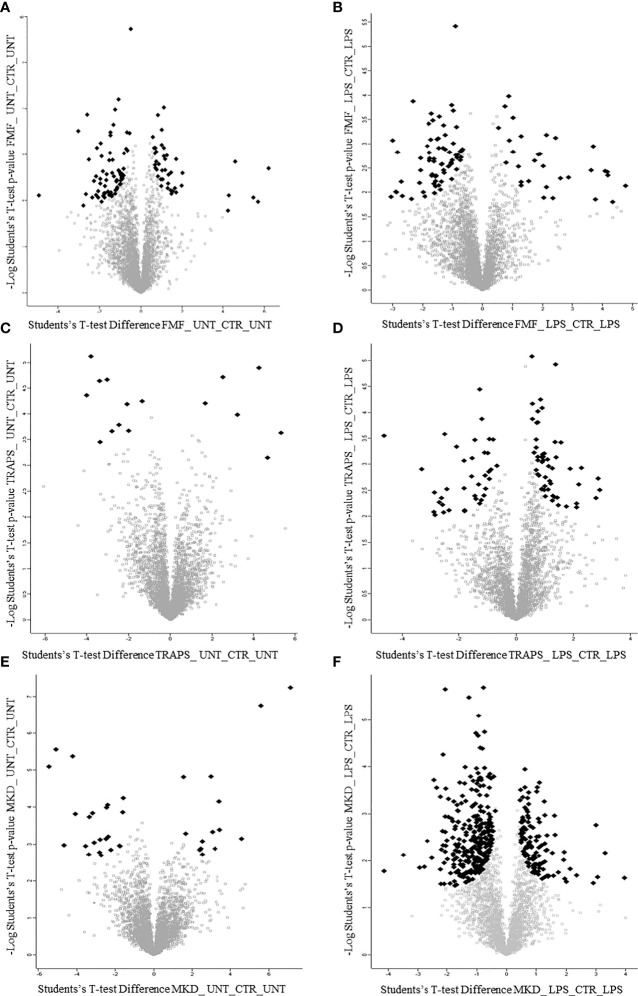
Volcano plots show proteins of untreated and LPS-treated monocytes of patients with FMF **(A, B)**, TRAPS **(C, D)** and MKD **(E, F)**. Black dots represent proteins with large magnitude fold-changes (x-axis) and high statistical significance (y-axis; p = −log10).

Principal component analysis (PCA) reduces the large number of variables (in our case proteins and their abundance) and describes each sample to a smaller number of latent variables. In this way we display differences and similarities between groups (**Figure 1D**). When comparing the subgroups for each disease, using t-test, we found that 114 and 99 proteins were significantly modulated in unstimulated and LPS-stimulated monocytes from FMF patients, respectively (**Figures 2A, B**; **Supplementary Tables S1**, **S2**). In TRAPS, proteins significantly modulated in unstimulated and LPS-stimulated monocytes were 15 and 88, respectively (**Figures 2C, D**; **Supplementary Tables S3**, **S4**). In MKD 35 proteins were specifically modulated in unstimulated monocytes and 413 in LPS-stimulated cells (**Figures 2E, F**; **Supplementary Tables S5**, **S6**).

Since LPS stimulation maximizes the differences in hyper-regulated proteins in monocytes in both the HRF and HDs groups, reducing the variability due to or pathological activity and/or the effects of ongoing treatment, we try to simultaneously visualize the information with a scatterplot of the fold change. For each condition we report the changes in the protein expression of stimulated diseased and healthy monocytes, normalized with their own control. Mathematically, the graph, **Supplementary Figure S1**, shows on a Cartesian plane the t-test fold change of the HRF-LPS/HRF-CTR pair against the fold change of HD-LPS/HD-CTR. Each of the 4 quadrants highlights, as described in results, the properties of each group.

### Disease-Specific Proteomic Signatures and Interactive Protein Network

Omics data visualization helps the understanding of complex, multidimensional data and allows filtering and highlighting the most important molecular elements associated with a given disease. To this aim we used two tools to interpret complex biological data: STRING and ClueGO. (see online **Supplemental Methods**).

**Figure 3:**
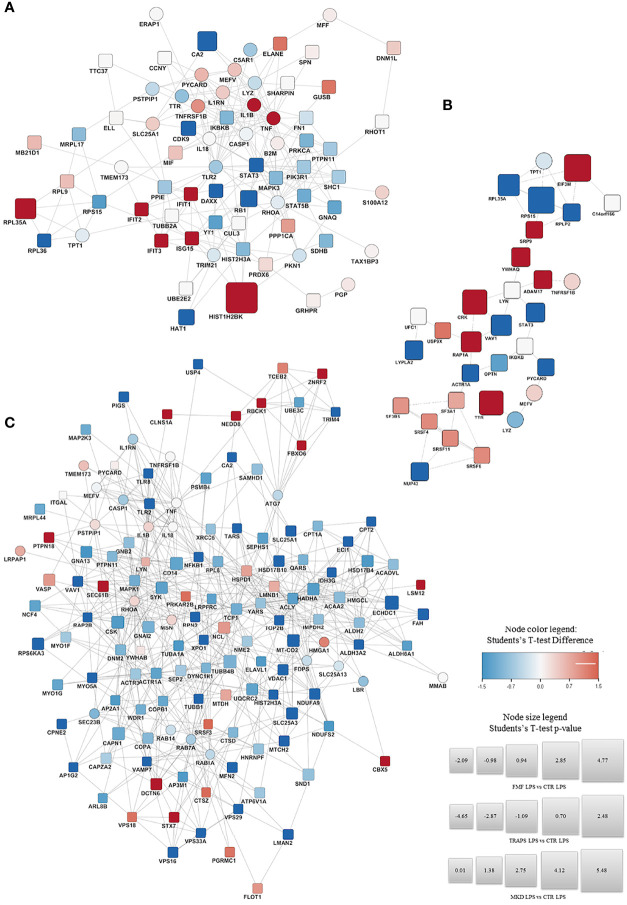
STRING Protein-protein interaction analyses. STRING PPI network connectivity on differential expressed proteins of the different Hereditary Recurrent Fevers integrated with DISEASE annotation of FMF, MVK, and TRAPS. Interaction network of proteins significantly up- (red) or down- regulated (blue) in LPS-treated monocytes of FMF **(A)**, TRAPS **(B)**, and MKD **(C)** patients compared with HD. Circle: proteins already known to be related to the diseases. Square: new proteins not yet related with the disease The size of each node expresses the percentage of protein concentration.

**Figure 3**; **Supplementary Figure S1** shown the proteins significantly up-regulated (in red) or down- regulated (in blue) in LPS-treated and untreated monocytes respectively for each HRF in respect to HD and their functional interactions analyzed with STRING protein-protein interaction analyses integrated with DISEASE annotation of FMF, MVK, and TRAPS.

#### FMF Proteomic Signature

In respect to HDs, LPS-stimulated FMF monocytes (**Figure 3A**) showed an up-regulation of IL-1β, TNFα and proteins associated with the interferon (IFN) activation, such as IFIT1, IFIT2, IFIT3, and the ubiquitin-like protein ISG15. The 60S ribosomal protein K related to protein transcription was also over-expressed. A down-regulation was observed for: STAT3 and CDK9 both related to the regulation of cytokines transcription; the 60S ribosomal protein RPL36, a component of the large ribosomal subunit; the histone acetyltransferase HAT1; the transcriptor modulator DAXX; the regulator of cell division RB1 and the carbon anhydrase CA2. Similarly, RhoA and various other proteins related to RhoA pathway (FN1, PKN1, GNAQ, and PTPN11) were down-regulated. The full list of the proteins over- and down-modulated is available in **Supplementary Table S7**.

The analysis of the interaction networks of LPS-stimulated monocytes from FMF patients (**Supplementary Figure S3**) showed that the main activated pathways were the advanced glycation end-products, the AGE-RAGE pathway and the Ras-related C3 botulinum toxin substrate 1 (RAC1) pathway. The signaling pathways related to different cytokines (IL-2/3/4/5/6/7/9/11) and type I IFN, were also also activated (**Supplementary Figure S3**). Additional activated pathways involved adipocytokine, leptin, prolactin, alpha 6 beta 4 integrin, thymic stromal lymphopoietin (TSLP), platelet-derived growth factor (PDGF), met, oncostatin M, erythropoietin (EPO) receptor and kit receptor.(**Supplementary Table S2**) Interestingly, the pathways associated with the regulation of the microtubule cytoskeleton, heat shock proteins and Fas ligand were also up-regulated.

#### TRAPS Proteomic Signature

The monocytes stimulated with LPS from TRAPS patients (**Figure 3B**) displayed an up-regulation of: ADAM17 and TNFRSF1B both associated with the TNF pathway. The same was for transthyretin (TTR) and 14-3-3 protein theta (YWHAQ), involved in the nucleocytoplasmic transport and Rho GTPase modulation, associated with the Pyrin-Inflammasome-; CRK and RAP1A, related to cell junction formation and GTPase activity. Proteins targeting secretory proteins, such as SRP9 or involved in the initiation of photosynthesis, like factor EIF3M were also up-regulated. Conversely, a down-regulation of STAT3; VAV1, a regulator of the Rho/Rac GTPases activation; and protein involved in microtubule stabilization and vesicle transport (ACTR1A, NUP43, TPT1) were observed. The full list is shown in **Supplementary Table S8**.

The main cellular pathways activated in LPS-stimulated monocytes from TRAPS patients were associated with the RNA splicing and transport, the inflammatory antiviral response, chemokine signaling, and leukocytes activation and degranulation (**Supplementary Figure S4**).

#### MKD Proteomic Signature

LPS-stimulated monocytes from MKD patients (**Figure 3C**), displayed a significant up-regulation of proteins involved in the regulation of the Pyrin Inflammasome such as RhoA, PSTPIP1, 14-3-3 protein beta/alpha (YWHAB) and Pycard. Other up-regulated proteins were PTPN18, NEDD8, FBXO6, LSM12 and TCEB2 that are involved in the polyubiquitination and proteasomal degradation. Furthermore, three ubiquitin-protein ligases (RANBP2, RBCK1, and ZNRF2) were also up-regulated. The same was observed for STX7, CLNS1A, DCTN6, SEC61B and VPS18, which are related to cellular trafficking. Also the chromobox CBX5 that binds histone H3 tails, the regulator of innate and adaptive immune response LYN, and FLOT1, acting as scaffold protein, were up-regulated.

Conversely, a down-regulation was observed for: NFKB1 and TLR2/8, related to the inflammatory response; GNA13, that activates Rho signaling; TRIM, E3 ubiquitin-protein ligase; LMAN2, AP1G2, MYO5A, RAB14 and RAB7A that are all involved in cellular trafficking; SYK, that phosphorylates the proto-oncogene VAV1; RAP2B, a GTP-binding protein; the different subunits VPS16/29/33A that constitute both the CORVET and HOPS complexes. Also proteins involved in the nuclear export (XPO1) or major constituent of microtubules (TUBB); or involved in regulation of actin filament disassembly (WDR1, ACTR3). Similarly, proteins involved in glycosylation (ACTR1A, RPN2) and fatty acid metabolism and mitochondrial function (CPT1A, CPT2, ECI1, and others; see **Supplementary Table S9**) were also down-modulated.

The main cellular pathways involved in LPS-stimulated monocytes in MKD patients were associated with the intermediate metabolism of glucose and other carbohydrates, to glycoprotein VI-mediated activation cascade and adaptive and innate immune response. Moreover, some pathways associated with the inflammatory response were also activated, such as the recycling pathways of IL-1, the NOD signaling, the NLRP3-inflammasome, and interleukin signaling (**Supplementary Figure S5**).

## Discussion

In this study, we provide the first report on the proteomics data analysis of unstimulated and LPS-stimulated monocytes of the three main monogenic HRFs compared to HDs. The major aim of the study was to identify a “proteomic signature” of unstimulated and LPS-stimulated monocytes for each genetic condition that might be considered as a possible biomarker for this condition. The study prompted the detection of specific proteins differentially expressed in the three HRFs, and the identification of possible unexplored intracellular pathways involved in the cellular response upon LPS stimulation. The choice to focus mainly on the LPS-stimulated monocytes was related to the need to overcome possible confounding factors related to a different disease activity or to the ongoing treatments at the moment of the sampling.

In LPS-treated FMF monocytes, the over-expression of IL-1β and TNFα is in line to their role as therapeutic targets in this condition. Interestingly, proteins related to the type 1 interferon pathway, such as IFIT1/2/3 and ISG15 are over-expressed in FMF (22–24). The lower expression of the IL-6-induced STAT3/CDK9 complex suggests a lower activation of the IL-6-mediated inflammatory response (25, 26). However, the possible increased oxidative stress in FMF patients is indicated by the higher concentration of the macrophage migration inhibitory factor (MIF) in unstimulated FMF monocytes with respect to HD (**Supplementary Table S7**) (27).

Other finely modulated proteins observed in LPS-stimulated FMF monocytes were CA2, the cell cycle regulators RB1 and DAXX, and the nuclear RPL35A, RPL36, HIST1H2BK and HAT. The up-regulation of CA2 is consistent with the efficacy of NSAIDs during FMF attacks (28). On the other hand, RB1 is the only protein dramatically reduced after LPS stimulation (**Supplementary Table S7**). Noteworthy, RB1 stabilizes microtubules-associated α-tubulin protein, suggesting that its loss may contribute to the cytoskeleton instability of FMF (29).

Similarly, the analysis of interaction networks highlights the abnormal regulation of pathways related to the chronic inflammation, microtubule cytoskeleton and cell apoptosis in FMF. The activation of the AGE-RAGE pathway triggers generation of free radicals and expression of pro-inflammatory gene mediators such as the SA100A12 that is highly expressed in the sera of FMF patients (30). Notably, the extracellular ligand of RAGE, S100A12, is also found up-regulated in untreated FMF monocytes (**Supplementary Table S7**), causing a dangerous feed-forward loop capable of shifting acute to chronic inflammation (31–33). This effect has been involved in many metabolic diseases, such as diabetes and insulin resistance, and contributes to modulate the signaling of adipocytokine, leptin, and prolactin, as endocrine disruptors (34, 35). A fascinating hypothesis poses the Mediterranean diet in defense against these secondary effects of the *MEFV* mutations (36). Several proteins and pathways related to cell proliferation and apoptosis can contribute to the significantly lower incidence of cancers in FMF patients (37).

In LPS-stimulated TRAPS monocytes, the up-regulation of the shedding protein ADAM17 suggests an ineffective attempt to correct the TNF receptor 1 shedding defect (38). TTR (Transthyretin) is an amyloidogenic protein and its overexpression in TRAPS patients could be associated with the possible evolution towards AA amyloidosis (39). Differently from FMF data, Pyrin-inflammasome is not up-regulated but even inhibited by the up-regulated 14-3-3 protein theta, a well-known pyrin inhibitor. Other modulated proteins in LPS-stimulated TRAPS monocytes are related to the translational machinery (SRP9, EIF3M and RPS15), vesicles transport (ACTR1A and NUP43) and mitogen-activated protein kinase pathway (LYPLA2, CRK, VAV1 and RAP1A). This evidence is in line with the hypothesis that a defect in the translation and/or transportation of the mutated TNF receptor 1 causes accumulation of the misfolded protein in the cytosol and Golgi, causing an unfolded-protein response (40–43). Interestingly, OPTN (Optineurin) seems partially reduced after LPS-stimulation, as well as the anti-inflammatory tyrosine kinase LYN, thus possibly inducing the stress of the endoplasmic reticulum and increasing autophagy during TRAPS-related inflammatory flares (**Supplementary Table S8**), as already described by our group (10, 44, 45).

MKD is a metabolic disease in which the defect of the pathway of cholesterol biosynthesis leads to a severe inflammatory phenotype. Among the three conditions analyzed, MKD displayed the highest level of complexity as far as the proteins regulation is concerned. Essentially, the pathways significantly over- or under-regulated in MKD are mainly involved in the intermediate metabolism, ubiquitination, intracellular trafficking, and inflammation. In particular, NEDD8, ZNRF2, FBXO6, LSM12 and HOIL-1, mediate protein ubiquitination. It is therefore possible that also the ubiquitination of inflammasomes may be affected (46). Other proteins, such as SEC61B, STX7, DCTN6, AP1G2, TUBB1, MYO5A, LMAN2, VPS16/29/33A and CPNE2 are generally associated with the regulation of the intracellular trafficking that seems to play an emerging role as trigger of autoinflammation (47, 48). Interestingly, PSTPIP1 whose mutations cause the PAPA syndrome and has a functional relationship with Pyrin, and its binding protein PTPN18 were up-regulated in MKD monocytes (**Supplementary Table S9**) (49). Finally, the up-regulated CBX5, also known as haptoglobin 1 alpha, have been associated with several inflammatory disorders due to its ability to modulate the helper T-cell type 1 and 2 balance with antioxidant properties (50).

The down-regulated proteins in the LPS-treated MKD monocytes are mainly associated to the NFkB-mediated inflammatory response (NFKB1, TLR2/8, RAP2B and TRIM4), as well as the DNA transcription and RNA nuclear export (CLNS1A, TOP2B, TARS and XPO1). This observation might explain the reduced microRNA-mediated silencing complex activity of the mevalonate pathway (51). This evidence might be associated with the increased susceptibility to infections observed in MKD patients, at variance with the other monogenic autoinflammatory diseases. Interestingly, several down-regulated proteins in MKD participate to the fatty acid oxidation and glycolipid biosynthesis (PIG-S, ALDH3A2, RPN2, CPT2, ECI1) supporting the existence of a prenylation defects of small GTPases in MKD (7, 52). Finally, the modulated MT-CO2, MFN2, MTCH2, IDH3G, NDUFA9, VDAC1, ECHDC1 and SLC25A1/3 proteins confirmed previous evidences of an unstable mitochondrial membrane and altered autophagy regulation in MKD monocytes (53).

The present study represents the first attempt of an unbiased analysis of the protein synthesis by stimulated monocytes derived from patients with the three main HRFs. Our results suggest the existence of a specific “proteomic signature” that could be of help in the identification of each condition. Moreover, cellular proteomic analysis is able to dissect different intracellular pathways involved in the cellular response upon a pro-inflammatory stimulus, indicating new possible unexplored pathways involved in the pathogenesis of each HRFs. Due to its exploratory nature, the present study was not aimed to verify the actual pathogenic impact of each protein or pathway through specific functional studies. In the discussion we have outlined only some of the possible functional interactions possibly related to what already known on the pathogenesis of each condition. These data can be also used in future to compare HRFs with non-genetic and multifactorial recurrent fevers, such as a multifactorial syndrome characterized by periodic fever, aphthous stomatitis, pharyngitis and adenopathy (PFAPA), and Syndrome of undifferentiated recurrent fever (SURF, an,heterogeneous group of autoinflammatory diseases characterized by self-limiting episodes of systemic inflammation without a confirmed molecular diagnosis) This comparison could allow identifying pathways involved in the pathogenesis of these multifactorial diseases and unravel a possible degree of overlap with other monogenic diseases.

## Data Availability Statement

The datasets presented in this study can be found in online repositories. The name of the repository and accession number can be found below: ProteomeXchange Consortium *via* the PRIDE partner repository, accession number PXD024068.

## Ethics Statement

The studies involving human participants were reviewed and approved by the Ethics Committee of the Gaslini Institute for biobanc “Istituzione di biobanca per pazienti affetti da malattie reumatiche dell’età pediatrica”: data approval 06/05/2004. Written informed consent to participate in this study was provided by the participants’ legal guardian/next of kin.

## Author Contributions

FP, conception and design, cells experiments, data collection and analysis, manuscript writing, and critical revision. AP, conception and design, Mass Spectrometry experiments, data collection and analysis, and critical revision. CL, Mass Spectrometry experiments, data collection and analysis. RP and SV, manuscript writing and critical revision. AB, data collection and analysis. AO, conception and design, patients’ selection, and clinical data collection. IG, MF and RC patients’ selection and clinical data collection. MG, conception and design, patients’ selection, manuscript writing, critical revision, and financial support. All authors contributed to the article andapproved the submitted version.

## Funding

This work was supported by the E-rare-3 project (INSAID, grant number 003037603) and by Ricerca Corrente Ministeriale of the Italian Health Ministry.

## Conflict of Interest

FP reports speaker fee from SOBI. MG reports grants and personal fees from Novartis, grants and personal fees from SOBI, outside the submitted work. RP reports speaker fee from SOBI. AB reports speaker fee from SOBI. SV reports speaker fee from SOBI. RC reports speaker fee from SOBI.

The remaining authors declare that the research was conducted in the absence of any commercial or financial relationships that could be construed as a potential conflict of interest.

## Publisher’s Note

All claims expressed in this article are solely those of the authors and do not necessarily represent those of their affiliated organizations, or those of the publisher, the editors and the reviewers. Any product that may be evaluated in this article, or claim that may be made by its manufacturer, is not guaranteed or endorsed by the publisher.

## Supplementary Material

Supplementary Figure 1 Volcano plots show up-regulated proteins of FMF, TRAPS, and MKD monocytes compared to HD in both steady state **(A, C, E)** and LPS treated **(B, D, E)**. Filled black diamond’s indicate statistically significant proteins that exceed the threshold of FDR<0.05 and S0>0.1, in contrast empty gray squares are proteins that were not significantly modulated and not considered in subsequent pathways and protein-protein network analyses.

Supplementary Figure 2 Interaction network of proteins significantly up- (red) or down- regulated (blue) in untreated monocytes of FMF **(A)**, TRAPS **(B)**, and MKD **(C)** patients compared with HD. Circle: proteins already known to be related to the diseases. Plot: new proteins not yet related with the disease The size of each node expresses the percentage of protein concentration.

Supplementary Figure 3 Hereditary recurrent fevers pathways overexpressed. Functional pathways networks upregulated in HRF in response to stimulus. ClueGO Cytoscape visualizes the interactions of protein clusters in a functionally grouped network using enrichment maps. The size of the nodes reflects the statistical significance of the terms. Only p-values < 0.05 are shown. For grouping terms, the initial group size was set to 2 and the percentage for merging groups was set to 20%. Groups were colored differently, red is HRF and Blue is HD. The ClueGO enrichment results illustrate that HRF modifies the metabolic behavior of signaling pathways, contributing to the manifestation of autoinflammatory disease with a group-specific signature. Furthermore, the dysregulated pathways identified by the enrichment analysis could show important roles in the pathogenesis of HRF. Interaction pathway network of untreated (blue) and LPS-treated (red) FMF monocytes. The size of each node expresses the degree of network activation.

Supplementary Figure 4 Interaction network of untreated (blue) and LPS-treated (red) TRAPS monocytes. The size of each node expresses the degree of network activation.

Supplementary Figure 5 Interaction network of untreated (blue) and LPS-treated (red) MKD monocytes. The size of each node expresses the degree of network activation.

Supplementary Table 1 Significantly modulated proteins of untreated FMF monocytes shown in **Figure 2A**.

Supplementary Table 2 Significantly modulated proteins of LPS-treated FMF monocytes shown in **Figure 2B**.

Supplementary Table 3 Significantly modulated proteins of untreated TRAPS monocytes shown in **Figure 2C**.

Supplementary Table 4 Significantly modulated proteins of LPS-treated TRAPS monocytes shown in **Figure 2D**.

Supplementary Table 5 Significantly modulated proteins of untreated MKD monocytes shown in **Figure 2E**.

Supplementary Table 6 Significantly modulated proteins of LPS-treated MKD monocytes shown in **Figure 2F**.

Supplementary Table 7 Significantly modulated proteins of untreated and LPS-treated FMF monocytes shown in **Figure 3A** and **Supplementary Figure 1A**, respectively.

Supplementary Table 8 Significantly modulated proteins of untreated and LPS-treated TRAPS monocytes shown in **Figure 3B** and **Supplementary Figure 1B**, respectively.

Supplementary Table 9 Significantly modulated proteins of untreated and LPS-treated MKD monocytes shown in **Figure 3C** and **Supplementary Figure 1C**, respectively.
