# Supplementary figures and images for: Proteomic Signatures of Monocytes in Hereditary Recurrent Fevers
Source: Front Immunol. 2022 Jun 23;13:921253. doi: 10.3389/fimmu.2022.921253 (PMC9260596; doi:10.3389/fimmu.2022.921253)

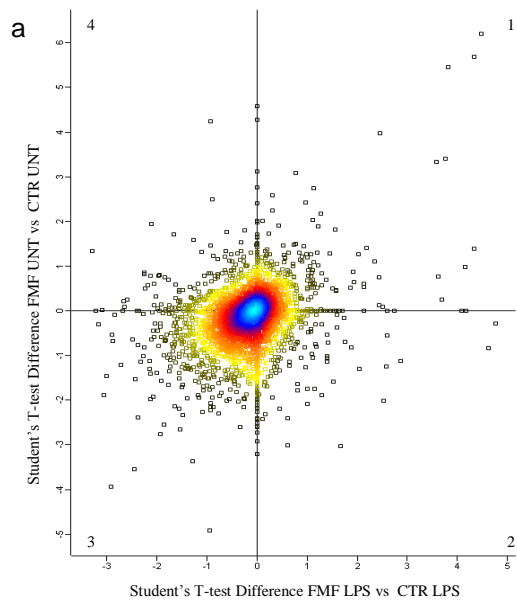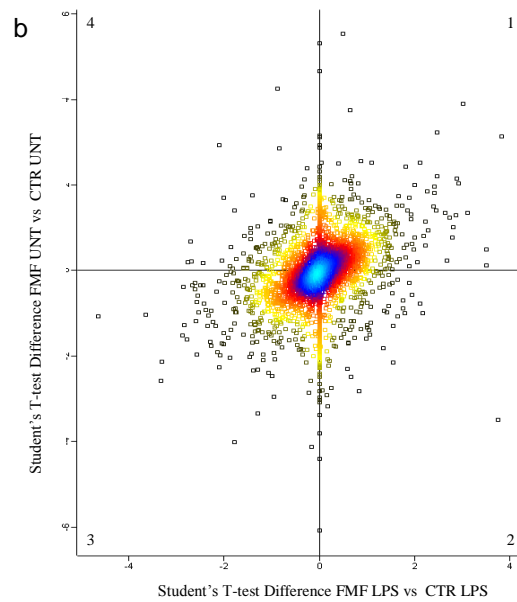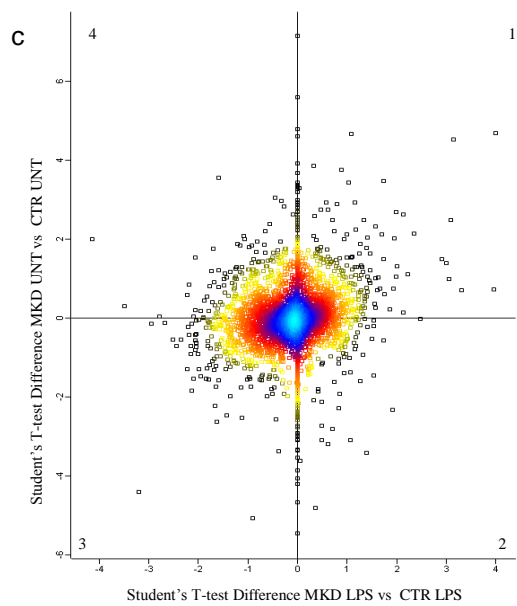



a

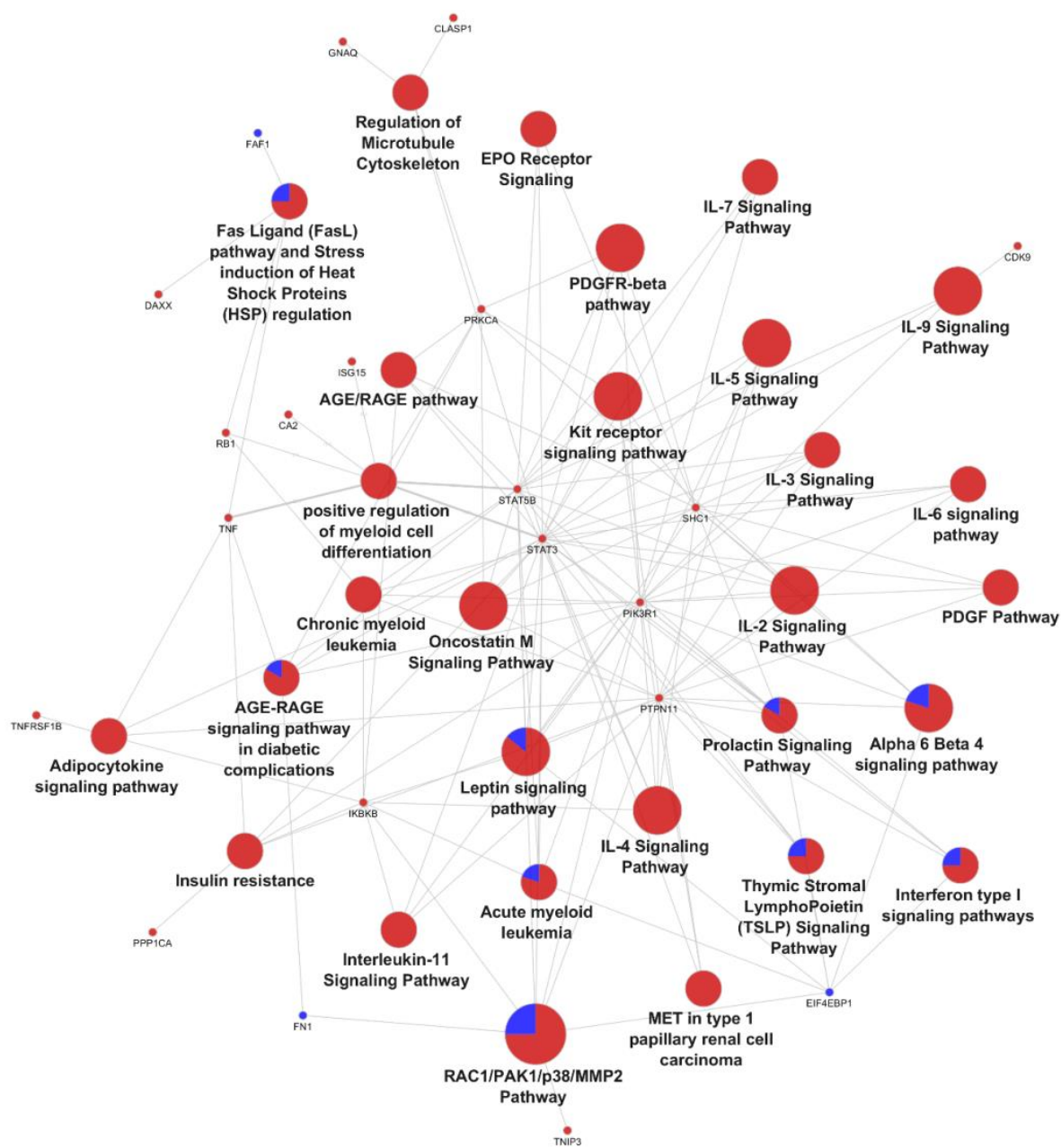

a

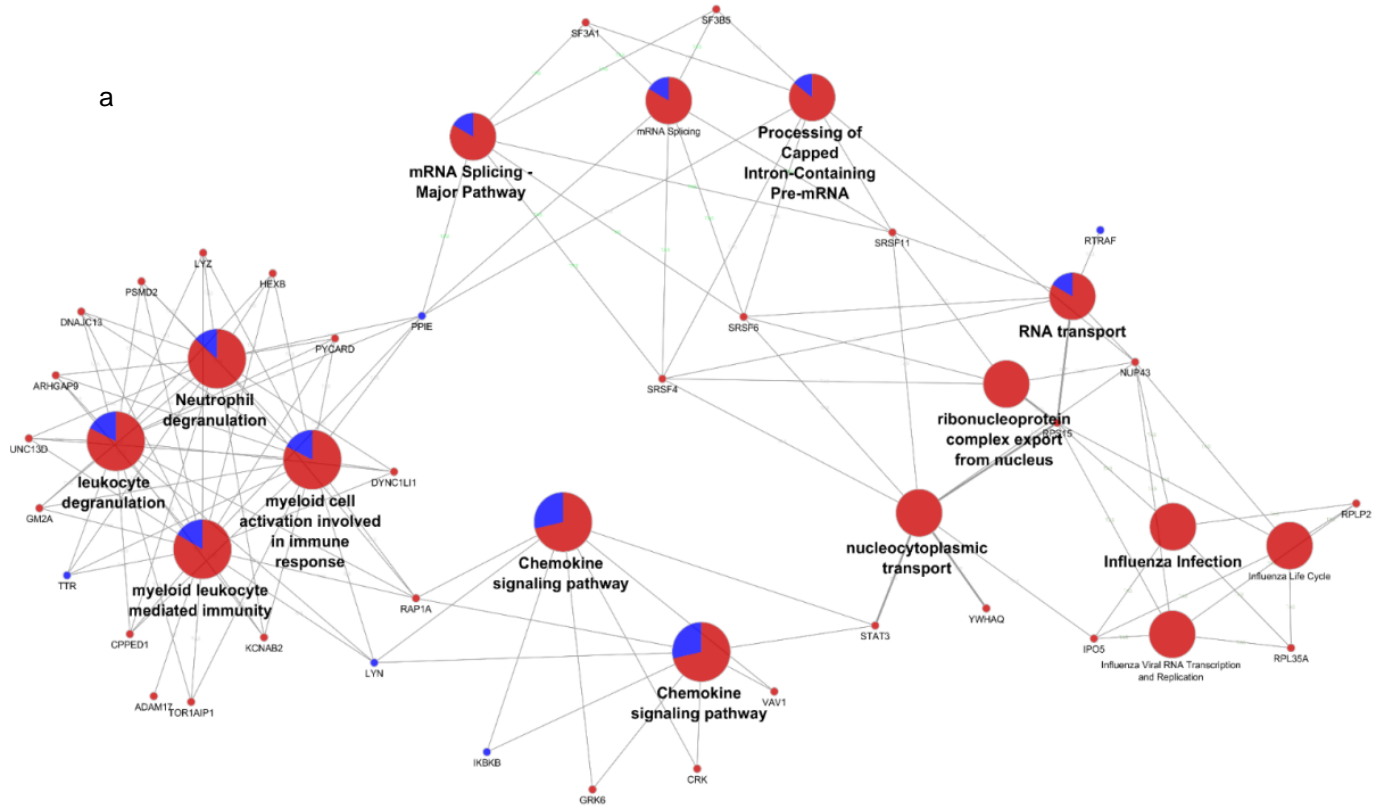

a

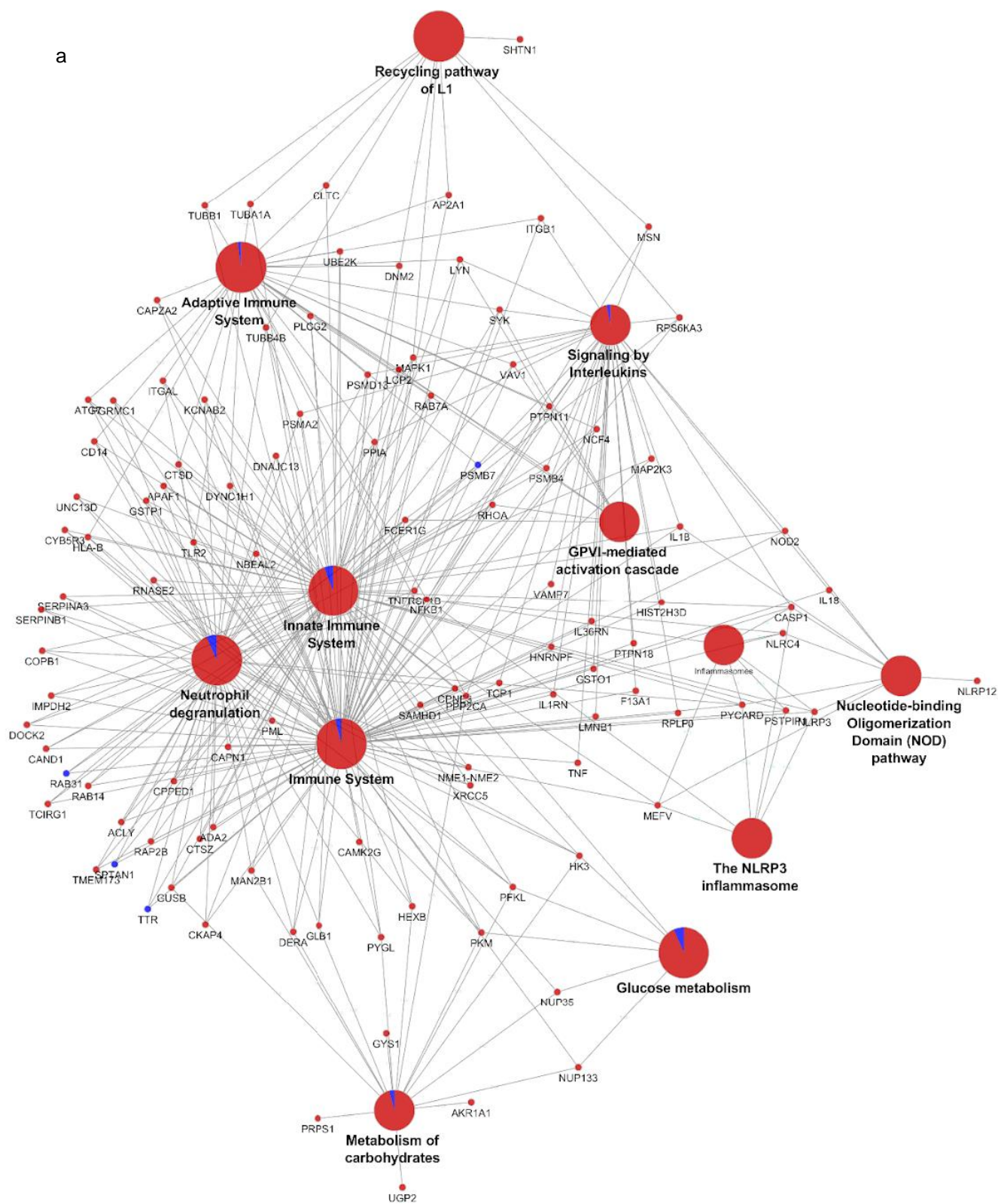

Supplement: Supplementary Figure 1 — Volcano plots show up-regulated proteins of FMF, TRAPS, and MKD monocytes compared to HD in both steady state (A, C, E) and LPS treated (B, D, E). Filled black diamond’s indicate statistically significant proteins that exceed the threshold of FDR<0.05 and S0>0.1, in contrast empty gray squares are proteins that were not significantly modulated and not considered in subsequent pathways and protein-protein network analyses. [file DataSheet_1.pdf]
