# Supplementary material for: Proteomic Signatures of Monocytes in Hereditary Recurrent Fevers
Source: Front Immunol. 2022 Jun 23;13:921253. doi: 10.3389/fimmu.2022.921253 (PMC9260596; doi:10.3389/fimmu.2022.921253)
Supplement: Supplementary Table 1 — Significantly modulated proteins of untreated FMF monocytes shown in Figure 2A. [file DataSheet_2.pdf]

Online Repository Tables

Table S1			
**Protein names**	**Gene names**	**Student's T-test p-value (-log)**	**Student's T-test Difference**
Translation initiation factor eIF-2B subunit delta	EIF2B4	2,6491	-1,08
BCL2/adenovirus E1B 19 kDa protein-interacting protein 3-like	BNIP3L	2,64253	-1,04628
Proteasomal ubiquitin receptor ADRM1	ADRM1	3,42823	-1,45877
Thromboxane-A synthase	TBXAS1	2,14246	-2,65853
Ubiquitin-protein ligase E3A	UBE3A	2,34873	-1,01414
Baculoviral IAP repeat-containing protein 1	NAIP	2,35951	-0,962915
Exosome complex component CSL4	EXOSC1	3,25102	-1,94119
von Willebrand factor A domain-containing protein 9	VWA9	3,21249	1,0017
Myeloid-associated differentiation marker	MYADM	2,46876	-0,901073
COP9 signalosome complex subunit 1	GPS1	2,57068	-1,6512
Actin beta	ACTB	1,78203	4,23435
Lactotransferrin	LTF	1,97549	5,68489
Serine/threonine-protein kinase MARK2	MARK2	2,1941	-1,57728
3-phosphoinositide-dependent protein kinase 1	PDPK1	2,29002	-1,1278
DNA (cytosine-5)-methyltransferase 3A	DNMT3A	2,88017	-1,36095
Translocation protein SEC62	SEC62	2,69098	-0,83733
Protein transport protein Sec24C	SEC24C	3,09638	-0,6466
Sjoegren syndrome/scleroderma autoantigen 1	SSSCA1	2,66599	1,16753
Cathelicidin antimicrobial peptide	CAMP	2,11231	4,27064
28S ribosomal protein S7, mitochondrial	MRPS7	2,15092	-1,50467
ORM1-like protein 3	ORMDL3	2,12002	-1,40518
Actinin alpha 4	ACTN4	2,06711	-2,05933
Transmembrane protein 205	TMEM205	2,42134	-2,26277
Dynamin-1-like protein	DNM1L	2,31057	-1,73744
Histone acetyltransferase type B catalytic subunit	HAT1	1,88924	-2,76738
2-deoxynucleoside 5-phosphate N-hydrolase 1	DNPH1	2,31399	0,988897
Syntaxin-6	STX6	2,06827	-2,33611
Uncharacterized protein KIAA0513	KIAA0513	2,1456	-1,23255

Galactosylgalactosylxylosylprotein 3-beta-glucuronosyltransferase 3	B3GAT3	2,22009	-1,21531
Thioredoxin domain-containing protein 12	TXNDC12	2,84474	0,747147
Fibronectin	FN1	3,87831	-2,59544
Non-histone chromosomal protein HMG-14	HMGN1	2,56373	-0,967116
Prosaposin	PSAP	2,79447	0,875078
Annexin A3	ANXA3	2,0623	5,45663
Macrophage migration inhibitory factor	MIF	2,60382	1,46934
Leukosialin	SPN	2,59416	2,01043
High mobility group protein HMG-I/HMG-Y	HMGA1	2,41716	-1,1831
Diacylglycerol kinase alpha	DGKA	2,87246	-1,53102
Peroxiredoxin-6	PRDX6	2,77405	0,686326
60S ribosomal protein L9	RPL9	3,02732	0,67707
Prohibitin	PHB	3,38393	0,576388
Replication factor C subunit 5	RFC5	2,15914	-1,77835
Cysteine--tRNA ligase, cytoplasmic	CARS	3,65283	-1,31223
RNA-binding protein 25	RBM25	3,0307	-0,811191
Ras-related protein Rab-9A	RAB9A	3,98751	-1,23249
RNA polymerase II elongation factor ELL	ELL	2,09464	-1,41141
Methionine--tRNA ligase, cytoplasmic	MARS	2,12011	-1,51632
ATP synthase subunit e, mitochondrial	ATP5I	2,46174	1,055
40S ribosomal protein S15	RPS15	2,60154	-0,989971
Histone H3.2	HIST2H3A	2,11215	-4,91382
Mitochondrial 2-oxoglutarate/malate carrier protein	SLC25A11	2,25039	-1,14861
Sialic acid-binding Ig-like lectin 14	SIGLEC14	2,19903	-2,05611
Heat shock protein 75 kDa, mitochondrial	TRAP1	2,53508	-0,936696
TAR DNA-binding protein 43	TARDBP	2,54596	-0,825465
Eukaryotic translation initiation factor 4E-binding protein 1	EIF4EBP1	3,06487	0,743391
Inositol-tetrakisphosphate 1-kinase	ITPK1	2,65068	1,03663
Cullin-3	CUL3	2,89467	-2,51483
Tubulin beta-2A chain	TUBB2A	2,10634	-1,80102
Nuclear transcription factor Y subunit gamma	NFYC	2,71195	0,712255

V-type proton ATPase subunit F	ATP6V1F	2,17341	1,71373
Putative coiled-coil-helix-coiled-coil-helix domain-containing protein CHCHD2P9, mitochondrial	CHCHD2P9	2,63145	-1,8274
Ubiquitin-associated protein 2	UBAP2	2,17427	-1,44638
Rootletin	CROCC	2,96526	1,10605
Vacuolar protein sorting-associated protein 53 homolog	VPS53	2,51984	-2,16291
Rap1 GTPase-activating protein 2	RAP1GAP2	2,36566	1,31747
Tetratricopeptide repeat protein 37	TTC37	2,12155	-2,16444
Vacuolar protein sorting-associated protein 13C	VPS13C	3,49152	-0,673022
UPF0317 protein C14orf159, mitochondrial	C14orf159	1,97671	-1,88973
Coiled-coil domain-containing protein 91	CCDC91	3,14619	-2,12756
Nitrilase homolog 1	NIT1	2,48105	-0,895555
Copine-8	CPNE8	2,15429	-1,72138
Cytokine receptor-like factor 3	CRLF3	2,42804	-0,981761
LysM and putative peptidoglycan-binding domain-containing protein 2	LYSMD2	2,56517	1,33936
Mitochondrial Rho GTPase 1	RHOT1	2,26671	1,64559
Misshapen-like kinase 1	MINK1	2,36364	-1,95205
Oxidation resistance protein 1	OXR1	2,4458	-1,16105
Eukaryotic translation initiation factor 4E type 3	EIF4E3	2,06687	-1,74517
Cyclic GMP-AMP synthase	MB21D1	3,51982	-3,01569
GTPase IMAP family member 8	GIMAP8	5,72817	-0,482056
Cyclin-Y	CCNY	2,32371	1,97999
ATP-binding cassette sub-family F member 1	ABCF1	2,48835	-0,929731
Retinol dehydrogenase 11	RDH11	2,10449	-1,42718
Nuclear pore membrane glycoprotein 210	NUP210	3,86408	0,818427
AP-3 complex subunit sigma-1	AP3S1	2,27	1,30312
Protein YIPF5	YIPF5	4,20425	-1,07156
Coiled-coil domain-containing protein 47	CCDC47	2,65329	-1,0721
RalBP1-associated Eps domain-containing protein 1	REPS1	3,54157	1,00333
Copine-2	CPNE2	2,45587	0,974257

ERO1-like protein alpha	ERO1L	3,15015	1,10495
Zinc finger RNA-binding protein	ZFR	3,03535	-1,48686
Ubiquitin-conjugating enzyme E2 E2	UBE2E2	2,84301	4,58013
Calcium and integrin-binding protein 1	CIB1	2,43848	-1,66321
N-terminal Xaa-Pro-Lys N-methyltransferase 1	NTMT1	2,52139	0,823927
RNA-binding protein 4	RBM4	3,14836	0,634053
Sharpin	SHARPIN	2,26113	-1,16509
Phosducin-like protein 3	PDCL3	2,90454	1,1818
Translation initiation factor IF-3, mitochondrial	MTIF3	2,2022	1,54116
MIP18 family protein FAM96A	FAM96A	2,97977	1,25787
Calcyclin-binding protein	CACYBP	3,47277	-0,546935
Integrator complex subunit 7	INTS7	2,47123	-1,96085
Aurora kinase A-interacting protein	AURKAIP1	2,54637	-0,819901
Cell growth-regulating nucleolar protein	LYAR	2,96601	-1,88782
NADH dehydrogenase [ubiquinone] 1 alpha subcomplex assembly factor 4	NDUFAF4	3,49299	-1,45721
Core histone macro-H2A.2	H2AFY2	2,90307	1,70402
Glyoxylate reductase/hydroxypyruvate reductase	GRHPR	3,14723	0,703817
Protein NipSnap homolog 3A	NIPSNAP3A	2,5962	-1,38459
U6 snRNA-associated Sm-like protein LSm7	LSM7	2,14617	1,48891
Glucocorticoid modulatory element-binding protein 2	GMEB2	2,49792	-0,921182
DNA helicase INO80	INO80	2,58241	-0,919009
FAS-associated factor 1	FAF1	2,66964	0,754553
Peptidyl-prolyl cis-trans isomerase E	PPIE	3,13143	-0,697405
Sialic acid-binding Ig-like lectin 9	SIGLEC9	2,52277	-1,3022
WD repeat-containing protein 7	WDR7	4,03392	1,10474
Myeloblastin	PRTN3	2,21776	1,43862

Table S2			
**Protein names**	**Gene names**	**Student's T-test p-value (-log)**	**Student's T-test Difference**
Apolipoprotein C-II	APOC2	2,65529	1,73212
AP-2 complex subunit mu	AP2M1	2,70471	-2,07333
Ig kappa chain C region	IGKV3-11	2,96108	-1,84914
Transmembrane emp24 domain-containing protein 7	TMED7-TICAM2	3,15646	1,2845
Killer cell lectin-like receptor subfamily F member 1	KLRF1	2,42047	4,14973
Thromboxane-A synthase	TBXAS1	2,34402	-1,54743
Phosphatidylinositol 3-kinase	PIK3C3	2,45059	-2,0696
Basic leucine zipper and W2 domain-containing protein 1	BZW1	3,07285	-3,00868
Myeloid-associated differentiation marker	MYADM	2,3412	-1,68624
COP9 signalosome complex subunit 1	GPS1	2,65123	-1,78145
G protein subunit beta 2	GNB2	2,54111	2,01711
Protein FAM195B	FAM195B	3,41629	-1,7937
Lactotransferrin	LTF	1,8043	4,34023
Guanine nucleotide-binding protein G(I)/G(S)/G(T) subunit beta-3	GNB3	2,79654	1,91601
2,5-phosphodiesterase 12	PDE12	2,60798	-1,04542
Aminoacyl-tRNA synthetases	WARS	3,12091	2,4317
Spermatogenesis-defective protein 39 homolog	VIPAS39	3,48995	-1,63996
Translocating chain-associated membrane protein 1	TRAM1	2,66561	-0,725739
REST corepressor 1	RCOR1	2,00124	-2,87949
Cytochrome B5 type B	CYB5B	2,12077	-1,56355
Myelin basic protein	MBP	1,91818	-1,93226
ATP-dependent RNA helicase DDX39A	DDX39A	2,85283	-0,690964
Insulin-like growth factor 2 mRNA-binding protein 3	IGF2BP3	2,01345	-2,89959
Inhibitor of nuclear factor kappa-B kinase subunit beta	IKBKB	3,013	-0,974699
Cytochrome b-c1 complex subunit 8	UQCRQ	2,71608	-0,836841
Histone H2B type 1-K	HIST1H2BK	2,13071	4,77234
Mitochondrial import receptor subunit TOM70	TOMM70A	2,84649	-0,732833
NADH dehydrogenase [ubiquinone] 1 alpha subcomplex subunit 3	NDUFA3	2,43581	4,07581
L-lactate dehydrogenase A chain	LDHA	3,77584	0,746957

Carbonic anhydrase 2	CA2	2,22256	-2,72336
Tumor necrosis factor	TNF	2,10769	2,12428
Ubiquitin-like protein ISG15	ISG15	2,30345	2,85696
Retinoblastoma-associated protein	RB1	2,19513	-2,1079
Epoxide hydrolase 1	EPHX1	2,82814	-1,09211
Beta-glucuronidase	GUSB	2,20811	1,2849
Neutrophil elastase	ELANE	2,1428	1,29188
Interferon-induced protein with tetratricopeptide repeats 1	IFIT1	1,88922	2,04699
Protein kinase C alpha type	PRKCA	2,27297	-1,02004
60S ribosomal protein L35a	RPL35A	2,95199	3,69147
Tumor necrosis factor receptor superfamily member 1B	TNFRSF1B	3,53611	1,02287
Succinate dehydrogenase [ubiquinone] iron-sulfur subunit, mitochondrial	SDHB	3,68361	-0,970757
Oxysterol-binding protein 1	OSBP	2,47088	-0,857971
Immunoglobulin alpha Fc receptor	FCAR	1,86006	-2,38474
Transcriptional repressor protein YY1	YY1	2,45033	-1,29518
Phosphatidylinositol 3-kinase regulatory subunit alpha	PIK3R1	2,72729	-0,716925
SHC-transforming protein 1	SHC1	2,88778	-0,660619
Enoyl-CoA hydratase, mitochondrial	ECHS1	3,98197	0,886471
ATP synthase subunit gamma, mitochondrial	ATP5C1	2,45458	3,61616
Signal transducer and activator of transcription 3	STAT3	2,69252	-1,61647
Guanine nucleotide-binding protein G(q) subunit alpha	GNAQ	3,18343	-1,22688
Cyclin-dependent kinase 9	CDK9	3,08943	-1,72175
Signal transducer and activator of transcription 5B	STAT5B	2,53191	-1,12713
Pterin-4-alpha-carbinolamine dehydratase	PCBD1	2,61119	0,791427
Serine/threonine-protein phosphatase PP1-alpha catalytic subunit	PPP1CA	3,06993	0,90153
U6 snRNA-associated Sm-like protein LSm6	LSM6	1,88535	2,34564
40S ribosomal protein S15	RPS15	2,58079	-1,33849
SUMO-conjugating enzyme UBC9	Ube2i	3,88061	-2,33522
Signal peptidase complex catalytic subunit SEC11A	SEC11A	2,88991	-1,23157
Neutrophil gelatinase-associated lipocalin	LCN2	1,85332	3,75308
Tyrosine-protein phosphatase non-receptor type 11	PTPN11	2,86577	-0,697657

Quinone oxidoreductase	CRYZ	2,82319	-2,84019
Mitochondrial-processing peptidase subunit alpha	PMPCA	2,40649	-1,27734
TAR DNA-binding protein 43	TARDBP	3,80335	-1,02279
Cytoskeleton-associated protein 5	CKAP5	2,77314	-0,818856
Putative heat shock protein HSP 90-alpha A4	HSP90AA4P	3,18752	2,13964
BRO1 domain-containing protein BROX	BROX	2,05345	-1,40511
Microtubule-associated protein 1S	MAP1S	2,9133	-1,2984
Type-1 angiotensin II receptor-associated protein	AGTRAP	1,98753	-1,59994
UPF0317 protein C14orf159, mitochondrial	C14orf159	1,91081	-3,06433
CLIP-associating protein 1	CLASP1	2,35413	4,17119
Putative phospholipase B-like 2	PLBD2	2,57368	-1,352
Tudor domain-containing protein 7	TDRD7	1,9284	-2,67805
Splicing factor U2AF 26 kDa subunit	U2AF1L4	2,29238	2,59262
Nuclear pore complex protein Nup133	NUP133	2,82081	-1,76479
Ubiquitin carboxyl-terminal hydrolase 7	USP7	3,38129	-1,2865
MOB kinase activator 3A	MOB3A	2,52959	1,19402
TNFAIP3-interacting protein 3	TNIP3	2,22684	1,54706
Retinoid-binding protein 7	RBP7	2,00695	-1,99031
Sorting nexin-18	SNX18	3,35005	-0,862465
Calcineurin B homologous protein 1	CHP1	3,56584	-1,433
Gamma-soluble NSF attachment protein	NAPG	2,17734	-1,61833
Death-inducer obliterator 1	DIDO1	2,5816	-1,03425
Protein PBDC1	PBDC1	2,66488	-1,00433
UBX domain-containing protein 6	UBXN6	2,36709	-1,66088
Rab3 GTPase-activating protein non-catalytic subunit	RAB3GAP2	2,63818	-1,61327
Rab GTPase-binding effector protein 2	RABEP2	2,22598	-1,54912
Complement component C1q receptor	CD93	2,6965	-1,76187
39S ribosomal protein L17, mitochondrial	MRPL17	5,41162	-0,887653
Structural maintenance of chromosomes protein 4	SMC4	2,22263	-1,35577
CKLF-like MARVEL transmembrane domain-containing protein 6	CMTM6	3,1136	-1,45964
ER membrane protein complex subunit 3	EMC3	2,91134	-1,45108

Protein RCC2	RCC2	3,3307	0,539761
COMM domain-containing protein 3	COMMD3	2,83432	1,0291
Death domain-associated protein 6	DAXX	2,32283	-1,55871
Ragulator complex protein LAMTOR2	LAMTOR2	2,78285	1,85818
WW domain-binding protein 11	WBP11	2,1449	-1,45081
ARF GTPase-activating protein GIT1	GIT1	2,76892	-0,881892
60S ribosomal protein L36	RPL36	3,10071	-1,50514
Mitochondrial carrier homolog 2	MTCH2	3,62523	-1,69682

Table S3			
**Protein names**	**Gene names**	**Student's T-test p-value (-log)**	**Student's T-test Difference**
U4/U6.U5 tri-snRNP-associated protein 2	USP39	4,64476	-3,37603
Beta-hexosaminidase	HEXA	4,36552	-4,00728
Inhibitor of nuclear factor kappa-B kinase subunit beta	IKBKB	3,78714	-2,44653
Glutaredoxin-3	GLRX3	4,24581	-1,35854
Transthyretin	TTR	4,71492	2,5282
Tyrosine-protein kinase Lyn	LYN	3,1506	4,66983
Ubiquitin-conjugating enzyme E2 K	UBE2K	4,66944	-3,01738
Eukaryotic translation initiation factor 3 subunit M	EIF3M	3,98755	3,2244
Parkinson disease 7 domain-containing protein 1	PDDC1	4,19438	-2,06145
Optineurin	OPTN	4,89887	4,25239
Type I inositol 3,4-bisphosphate 4-phosphatase	INPP4A	3,67887	-2,00061
39S ribosomal protein L39, mitochondrial	MRPL39	4,20496	1,66681
Peptidyl-prolyl cis-trans isomerase E	PPIE	3,66788	-2,80125
UPF0568 protein C14orf166	C14orf166	5,11778	-3,80364
Ubiquitin-fold modifier-conjugating enzyme 1	UFC1	3,4539	-3,37135

Table S4			
**Protein names**	**Gene names**	**Student's T-test p-value (-log)**	**Student's T-test Difference**
Acyl-coenzyme A thioesterase 1	ACOT1	2,91761	1,8944
Killer cell lectin-like receptor subfamily F member 1	KLRF1	2,35408	2,78495
Zinc finger Ran-binding domain-containing protein 2	ZRANB2	2,92689	2,29108
Ubiquitin-like modifier-activating enzyme 6	UBA6	2,34883	-2,60249
Phosphoglycolate phosphatase	PGP	3,87107	-1,19608
GTP-binding protein Rheb	RHEB	3,06775	-1,81305
Protein PRRC2C	PRRC2C	2,8109	0,757051
ATP synthase F1 subunit beta	ATP5B	3,34007	-2,09055
Serine/threonine-protein phosphatase	PPP4C	2,37124	1,35351
Serine/arginine-rich splicing factor 2	SRSF2	5,07821	0,553397
Sulfatase-modifying factor 2	SUMF2	2,50839	0,993273
Lon protease homolog, mitochondrial	LONP1	2,22189	-2,56644
Transcription initiation factor TFIID subunit 4	TAF4	2,52059	0,902915
Importin-5	IPO5	3,58239	-2,49514
Long-chain-fatty-acid--CoA ligase 4	ACSL4	2,33436	-1,4109
Syntaxin-10	STX10	2,39669	-1,44128
DnaJ homolog subfamily C member 13	DNAJC13	2,1122	-2,3428
Acyl-protein thioesterase 2	LYPLA2	2,1156	-1,79507
Apoptosis-inducing factor 1, mitochondrial	AIFM1	2,31319	-1,21197
Transthyretin	TTR	2,17034	2,11562
Superoxide dismutase [Mn], mitochondrial	SOD2	3,47564	-0,795899
60S acidic ribosomal protein P2	RPLP2	2,24316	-1,28098
Uroporphyrinogen decarboxylase	UROD	3,79867	0,718335
Beta-hexosaminidase subunit beta	HEXB	2,85845	-0,943622
Histone H2A.V	H2AFV	3,2165	-0,997572
Prolyl 4-hydroxylase subunit alpha-1	P4HA1	3,20409	0,991196
Proto-oncogene vav	VAV1	2,52521	-2,42122
Ganglioside GM2 activator	GM2A	2,90487	-3,32058
60S ribosomal protein L35a	RPL35A	2,02909	-2,83904

Oxysterol-binding protein 1	OSBP	2,92113	1,26053
DNA replication licensing factor MCM3	MCM3	2,76898	-1,55605
14-3-3 protein theta	YWHAQ	4,92329	1,3736
Proteasome subunit beta type	PSM8	3,48298	-0,942297
Signal transducer and activator of transcription 3	STAT3	2,54145	-1,81738
G protein-coupled receptor kinase 6	GRK6	3,42739	1,3553
Adapter molecule crk	CRK	2,61655	2,1844
Dolichyl-diphosphooligosaccharide--protein glycosyltransferase subunit STT3A	STT3A	2,26965	-2,68238
Signal recognition particle 9 kDa protein	SRP9	2,51648	1,1249
Rap1 GTPase-GDP dissociation stimulator 1	RAP1GDS1	3,31793	0,695963
Arginine--tRNA ligase, cytoplasmic	RARS	4,44209	-1,27305
Adenosine kinase	ADK	2,21462	1,47635
6.8 kDa mitochondrial proteolipid	MP68	2,5083	2,93227
Alpha-centractin	ACTR1A	2,40034	-1,12017
Lysozyme C	LYZ	2,97255	-0,677104
Ras-related protein Rap-1A	RAP1A	3,41596	1,57156
40S ribosomal protein S15	RPS15	3,55048	-4,64983
Disintegrin and metalloproteinase domain-containing protein 17	ADAM17	2,39435	1,27999
Tumor necrosis factor alpha-induced protein 2	TNFAIP2	2,08198	-2,86997
Serine/arginine-rich splicing factor 11	SRSF11	3,07982	0,768003
Serine/arginine-rich splicing factor 4	SRSF4	3,80635	0,766953
Syntaxin-5	STX5	3,04753	0,905947
26S proteasome non-ATPase regulatory subunit 2	PSMD2	4,24552	0,847585
Serine/arginine-rich splicing factor 6	SRSF6	4,02532	0,769634
Voltage-gated potassium channel subunit beta-2	KCNAB2	3,46974	-1,43573
Cold-inducible RNA-binding protein	CIRBP	2,74965	1,03243
Splicing factor 3A subunit 1	SF3A1	3,87414	0,572037
NADH dehydrogenase [ubiquinone] 1 alpha subcomplex subunit 5	NDUFA5	2,78074	-1,08153
Putative oxidoreductase GLYR1	GLYR1	3,1385	0,765809
Torsin-1A-interacting protein 1	TOR1AIP1	3,22079	0,621812
Ubiquitin-associated protein 2	UBAP2	3,14002	0,946587

Aftiphilin	AFTPH	2,29981	1,27546
Protein unc-13 homolog D	UNC13D	2,71693	-0,935266
COMM domain-containing protein 1	COMMD1	2,60889	-1,30781
Nucleoporin Nup43	NUP43	3,12088	-1,54425
TBC1 domain family member 15	TBC1D15	2,67981	1,16709
Serine/threonine-protein phosphatase 4 regulatory subunit 1	PPP4R1	3,14244	0,889703
TBC1 domain family member 5	TBC1D5	2,07304	-2,53172
La-related protein 4B	LARP4B	2,1023	-1,80506
Probable ubiquitin carboxyl-terminal hydrolase FAF-X	USP9X	4,08315	0,908494
Protein YIPF5	YIPF5	3,19344	0,935504
Mediator of RNA polymerase II transcription subunit 8	MED8	3,09076	1,09395
MMS19 nucleotide excision repair protein homolog	MMS19	2,19116	1,76638
Protein NipSnap homolog 1	NIPSNAP1	2,62543	1,20919
Serine/threonine-protein phosphatase CPPED1	CPPED1	2,96519	1,12858
Rho GTPase-activating protein 9	ARHGAP9	4,16595	0,576696
RNMT-activating mini protein	FAM103A1	3,08062	0,949156
N-terminal Xaa-Pro-Lys N-methyltransferase 1	NTMT1	2,88891	0,713439
Splicing factor 3B subunit 5	SF3B5	3,47688	0,699482
Chitinase domain-containing protein 1	CHID1	2,57685	1,00737
Protein phosphatase 1 regulatory subunit 12C	PPP1R12C	2,7828	0,802731
GTP-binding protein SAR1a	SAR1A	2,35959	1,43886
Mitochondrial import receptor subunit TOM22 homolog	TOMM22	2,38449	1,0312
Myoferlin	MYOF	2,45846	-2,84924
Apoptosis-associated speck-like protein containing a CARD	PYCARD	2,52971	-1,07121
Zinc finger protein 330	ZNF330	2,52607	0,902349
Hypoxia up-regulated protein 1	HYOU1	3,22441	-1,1079
Transforming acidic coiled-coil-containing protein 3	TACC3	3,13558	1,40283
Cytoplasmic dynein 1 light intermediate chain 1	DYNC1LI1	2,89763	-0,883852

Table S5			
**Protein names**	**Gene names**	**Student's T-test p-value (-log)**	**Student's T-test Difference**
A-kinase anchor protein 9	AKAP9	5,09991	-5,45275
COMM domain-containing protein 4	COMMD4	3,28256	1,655
von Willebrand factor A domain-containing protein 8	VWA8	3,1464	4,5966
Heat shock protein family A member 8	HSPA8	3,11882	-2,77979
HEAT repeat-containing protein 5A	HEATR5A	5,3754	-4,19927
Transmembrane and coiled-coil domain-containing protein 1	TMCO1	2,71126	2,52264
Ras-related protein Rab-4B	RAB4B	3,39498	3,42834
Fructose-1,6-bisphosphatase isozyme 2	FBP2	3,98947	-2,4602
Transthyretin	TTR	2,97015	-4,66502
Anion exchange protein 2	SLC4A2	3,86877	-1,61614
Spermidine synthase	SRM	2,71081	-3,37023
High mobility group nucleosome-binding domain-containing protein 5	HMGN5	3,20722	-2,36694
Aminoacylase-1	ACY1	4,24728	-1,58658
T-lymphoma invasion and metastasis-inducing protein 1	TIAM1	4,06037	-2,40614
Myotubularin-related protein 3	MTMR3	2,69437	-2,73138
Ras-related protein Rab-31	RAB31	4,81537	1,53276
Spectrin alpha chain, non-erythrocytic 1	SPTAN1	2,76857	-2,80785
Protein RRP5 homolog	PDCD11	4,82902	2,97891
Rootletin	CROCC	5,55965	-5,07579
Ninein	NIN	7,24404	7,13879
Tetratricopeptide repeat protein 9C	TTC9C	3,83862	-3,18524
Mitochondrial import inner membrane translocase subunit TIM14	DNAJC19	3,04649	-3,09963
Proteasome subunit beta type-7	PSMB7	3,33627	3,04137
CD180 antigen	CD180	3,74148	-3,34947
M-phase phosphoprotein 8	MPHOSPH8	2,88289	2,43691
Ubiquitin-like protein 5	UBL5	4,15996	3,42452
Mitochondrial fission factor	MFF	2,83858	2,37847

Kinesin-like protein KIF13A	KIF13A	2,95261	-3,54405
Olfactory receptor 10A2	OR10A2	2,84901	-2,22888
Conserved oligomeric Golgi complex subunit 4	COG4	2,87489	3,17967
Phosphopantothenate--cysteine ligase	PPCS	3,81922	-4,05984
Histidine triad nucleotide-binding protein 3	HINT3	3,14403	-2,48642
3-oxoacyl-[acyl-carrier-protein] synthase, mitochondrial	OXSM	2,95555	-1,79409
Protocadherin beta-15	PCDHB15	6,76271	5,59694
General transcription factor 3C polypeptide 5	GTF3C5	2,95287	-1,74294

Table S6			
**Protein names**	**Gene names**	**Student's T-test p-value (-log)**	**Student's T-test Difference**
40S ribosomal protein S9	RPS9	3,6641	-1,56307
Proteasome subunit alpha type	PSMA2	1,98229	-0,758658
Zinc finger protein 787	ZNF787	2,89637	1,0644
40S ribosomal protein S24	RPS24	2,43325	-1,99903
Clathrin heavy chain	CLTC	3,06215	-0,871793
GMP reductase	GMPR2	2,85874	-0,977707
NADH dehydrogenase [ubiquinone] 1 alpha subcomplex subunit 10, mitochondrial	NDUFA10	2,2165	-1,33103
ATPase ASNA1	ASNA1	1,95426	-0,795187
Selenoprotein H	C11orf31	3,29106	0,445317
Proteasome activator complex subunit 2	PSME2	1,73527	0,931124
Calcium/calmodulin-dependent protein kinase type II subunit gamma	CAMK2G	2,31833	-1,06501
Isovaleryl-CoA dehydrogenase, mitochondrial	IVD	1,72859	-1,19169
Integrin-linked protein kinase	ILK	2,62889	-0,740769
Sulfotransferase	SULT1A4	2,26674	-1,4465
UPF0693 protein C10orf32	C10orf32-ASMT	1,98054	1,27572
Serine/arginine-rich splicing factor 7	SRSF7	2,86164	0,583411
Dolichyl-diphosphooligosaccharide--protein glycosyltransferase 48 kDa subunit	DDOST	2,2896	-1,07467
DNA-directed RNA polymerase	POLR2A	3,52349	-1,16738
Thromboxane-A synthase	TBXAS1	3,55787	-2,35376
Fatty acid synthase	FASN	1,9973	-1,56089
Shootin-1	KIAA1598	2,02041	0,883891
Aconitate hydratase, mitochondrial	ACO2	2,64385	-0,568282
Small ubiquitin-related modifier 3	SUMO3	1,61329	1,95791
Pituitary tumor-transforming gene 1 protein-interacting protein	PTTG1IP	2,17257	1,53228
Unconventional myosin-Ig	MYO1G	2,56182	-0,850527
Polyadenylate-binding protein	PABPC4	3,38119	-1,486
Branched-chain-amino-acid aminotransferase	BCAT2	1,91725	-1,76094
Septin-11	11-set	1,72841	-1,04883
Annexin	ANXA6	2,49616	-0,943773

Protein PRRC2C	PRRC2C	2,91316	0,74127
Casein kinase II subunit alpha	CSNK2A1	2,93641	-0,547729
Complement receptor type 1	CR1	2,32872	-2,04296
Transducin beta-like protein 2	TBL2	2,31121	-0,550451
Transformation/transcription domain-associated protein	TRRAP	2,01142	1,9173
Protein transport protein Sec23A	SEC23A	2,95576	-1,05487
2,5-phosphodiesterase 12	PDE12	2,046	-0,666538
CAD protein	CAD	2,90084	-1,85107
Phosphatidylinositol 4,5-bisphosphate 3-kinase catalytic subunit delta isoform	PIK3CD	2,62517	-1,09182
Eukaryotic translation initiation factor 2A	EIF2A	2,45773	0,625928
Translocation protein SEC62	SEC62	2,18751	1,92992
Aminoacyl-tRNA synthetases	WARS	2,76214	3,00623
Sp110 nuclear body protein	SP110	1,51842	2,91582
AMP deaminase 2	AMPD2	2,13904	0,600659
Methyltransferase-like protein 7A	METTL7A	2,63543	-2,1536
RNA-binding protein with serine-rich domain 1	RNPS1	2,85446	0,561779
Beta-hexosaminidase	HEXA	1,95113	-2,08827
Proteasome-associated protein ECM29 homolog	KIAA0368	1,70311	-1,46049
REST corepressor 1	RCOR1	2,03264	0,763764
Ubiquitin thioesterase OTUB1	OTUB1	2,1114	-0,781033
Tubulin-specific chaperone D	TBCD	1,91019	-1,46345
Protein SSXT	SS18	1,92048	0,988246
Histone H3	H3F3B	2,12753	-3,47977
Lon protease homolog, mitochondrial	LONP1	1,77697	-1,1821
Transmembrane protein 205	TMEM205	1,7569	-1,23679
Mitochondrial import inner membrane translocase subunit TIM44	TIMM44	2,9368	-1,94859
Unconventional myosin-If	MYO1F	2,44848	-0,495675
Membrane-associated progesterone receptor component 1	PGRMC1	2,05226	0,858472
Dynactin subunit 6	DCTN6	3,56454	1,02879
High mobility group nucleosome-binding domain-containing protein 4	HMGN4	1,5482	1,99868
Lysosomal alpha-mannosidase	MAN2B1	3,21644	-1,05135

Fructose-1,6-bisphosphatase isozyme 2	FBP2	2,12745	-1,42789
Apoptotic protease-activating factor 1	APAF1	3,31953	-1,94591
Exportin-1	XPO1	1,95858	-1,28982
FYN-binding protein	FYB	1,93861	0,74564
Actin-related protein 2/3 complex subunit 3	ARPC3	1,78974	-0,900927
Serine palmitoyltransferase 1	SPTLC1	1,81264	-0,802845
Syntaxin-7	STX7	2,52298	1,08607
U4/U6 small nuclear ribonucleoprotein Prp3	PRPF3	2,22094	1,32621
Sjoegren syndrome nuclear autoantigen 1	SSNA1	1,78858	0,937857
A-kinase anchor protein 8	AKAP8	1,7539	1,7313
Isocitrate dehydrogenase [NAD] subunit beta, mitochondrial	IDH3B	3,15707	-1,1633
Intron-binding protein aquarius	AQR	1,87165	-2,78409
Procollagen-lysine,2-oxoglutarate 5-dioxygenase 3	PLOD3	1,50536	-2,09434
Toll-like receptor 2	TLR2	2,55182	-1,35848
H/ACA ribonucleoprotein complex subunit 4	DKC1	2,01105	-1,06335
WD repeat-containing protein 1	WDR1	1,97443	-0,770498
Copine-3	CPNE3	1,55489	-1,74597
DnaJ homolog subfamily C member 13	DNAJC13	2,36391	-1,6024
NADH dehydrogenase [ubiquinone] iron-sulfur protein 2, mitochondrial	NDUFS2	2,06543	-0,99331
Core histone macro-H2A.1	H2AFY	3,75721	-0,528851
NADH dehydrogenase [ubiquinone] iron-sulfur protein 3, mitochondrial	NDUFS3	3,06566	-1,62435
Interferon-inducible double-stranded RNA-dependent protein kinase activator A	PRKRA	1,67361	-1,5785
Acyl-protein thioesterase 1	LYPLA1	2,42778	-1,43566
U5 small nuclear ribonucleoprotein 200 kDa helicase	SNRNP200	3,70966	-0,9646
Eukaryotic translation initiation factor 3 subunit J	EIF3J	3,2084	0,74896
AP-1 complex subunit gamma-like 2	AP1G2	1,75285	-1,69973
CAAX prenyl protease 1 homolog	ZMPSTE24	1,85534	-1,77427
Flotillin-1	FLOT1	2,09824	0,637147
Glutaredoxin-3	GLRX3	1,75863	-1,28176
TOX high mobility group box family member 4	TOX4	1,76785	1,06289
Mitofusin-2	MFN2	2,60447	-1,35445

Reticulon-3	RTN3	1,89289	1,07761
Vesicle-associated membrane protein-associated protein B/C	VAPB	1,65561	1,39322
NAD kinase	NADK	1,51436	-1,90481
AP-2 complex subunit alpha-1	AP2A1	1,86978	-0,968019
Peroxisomal membrane protein 11B	PEX11B	2,01916	-1,77389
L-lactate dehydrogenase A chain	LDHA	2,39312	-0,764338
NADH-cytochrome b5 reductase 3	CYB5R3	2,49853	-1,81116
Cytochrome c oxidase subunit 2	MT-CO2	4,25766	-2,13599
Coagulation factor XIII A chain	F13A1	2,20862	-1,28347
Aspartate aminotransferase, mitochondrial	GOT2	3,79135	-1,20688
Carbonic anhydrase 2	CA2	1,78639	-1,62715
Alpha-1-antichymotrypsin	SERPINA3	2,21278	-0,619604
Dolichyl-diphosphooligosaccharide--protein glycosyltransferase subunit 2	RPN2	2,10215	-1,14633
Guanine nucleotide-binding protein G(i) subunit alpha-2	GNAI2	3,97126	-0,748285
Aldehyde dehydrogenase, mitochondrial	ALDH2	2,62104	-0,633041
60S acidic ribosomal protein P0	RPLP0	2,41834	-1,03796
Integrin beta-1	ITGB1	3,85059	-0,559403
Glycogen phosphorylase, liver form	PYGL	3,48884	-0,993727
Nucleophosmin	NPM1	2,82436	0,529038
Cathepsin D	CTSD	2,07031	-0,680798
Calpain-1 catalytic subunit	CAPN1	4,38378	-0,817368
Tubulin beta chain	TUBB	2,36384	-0,63584
Beta-hexosaminidase subunit beta	HEXB	2,28612	-1,46273
Tyrosine-protein kinase Lyn	LYN	1,78399	-1,02416
Tropomyosin beta chain	TPM2	1,64658	3,05169
Fumarate hydratase, mitochondrial	FH	2,46166	-0,662382
Annexin A6	ANXA6	3,76216	-0,862628
Beta-glucuronidase	GUSB	1,79523	-0,847849
Monocyte differentiation antigen CD14	CD14	4,40179	-0,876353
Annexin A5	ANXA5	4,71894	-1,03138
Glutathione S-transferase P	GSTP1	3,9671	-1,00345

Chromosome transmission fidelity protein 8 homolog isoform 2	CHTF8	2,36213	0,763079
Non-secretory ribonuclease	RNASE2	3,36359	-2,21522
Cytochrome c oxidase subunit 5B, mitochondrial	COX5B	1,87498	0,748083
60 kDa heat shock protein, mitochondrial	HSPD1	2,59983	0,559345
Histidine--tRNA ligase, cytoplasmic	HARS	1,85024	-1,05112
Inosine-5-monophosphate dehydrogenase 2	IMPDH2	2,17477	-0,604184
Xaa-Pro dipeptidase	PEPD	1,87983	-0,917921
X-ray repair cross-complementing protein 5	XRCC5	2,34754	-0,580843
Glycogen [starch] synthase, muscle	GYS1	1,88678	-0,991416
Hematopoietic lineage cell-specific protein	HCLS1	2,11451	0,722314
Alcohol dehydrogenase [NADP(+)]	AKR1A1	2,12813	-0,77733
Pyruvate kinase PKM	PKM	2,99865	-0,588267
Proto-oncogene vav	VAV1	2,49495	-1,09344
Carbonyl reductase [NADPH] 1	CBR1	2,88512	-0,574039
Beta-galactosidase	GLB1	2,20238	-0,907407
NADPH--cytochrome P450 reductase	POR	5,65244	-2,06288
1-phosphatidylinositol 4,5-bisphosphate phosphodiesterase gamma-2	PLCG2	2,09533	-0,627927
Fumarylacetoacetase	FAH	3,27055	-1,37436
Calpain-2 catalytic subunit	CAPN2	2,65472	-0,924127
ATP-dependent 6-phosphofructokinase, liver type	PFKL	2,37931	-0,72436
T-complex protein 1 subunit alpha	TCP1	3,04807	-0,777268
Nucleolin	NCL	3,95183	0,622529
Hexokinase-1	HK1	2,94551	-1,09912
Casein kinase II subunit alpha	CSNK2A2	2,14554	-0,821901
Nuclear factor NF-kappa-B p105 subunit	NFKB1	1,90645	-1,10802
Transcription factor BTF3	BTF3	2,318	0,827761
Lamin-B1	LMNB1	2,43947	0,495247
Integrin alpha-L	ITGAL	2,48374	-0,843106
Voltage-dependent anion-selective channel protein 1	VDAC1	2,86398	-1,27154
Nucleoside diphosphate kinase B	NME2	2,3839	-0,497314
Cytochrome b-c1 complex subunit 2, mitochondrial	UQCRC2	3,59401	-0,881662

Splicing factor, proline- and glutamine-rich	SFPQ	2,58002	0,475691
NAD-dependent malic enzyme, mitochondrial	ME2	2,23534	-0,596182
Carnitine O-palmitoyltransferase 2, mitochondrial	CPT2	1,79399	-1,19811
ATP synthase F(0) complex subunit B1, mitochondrial	ATP5F1	2,16714	-1,39646
ATP synthase subunit alpha, mitochondrial	ATP5A1	2,64284	-0,513721
Threonine--tRNA ligase, cytoplasmic	TARS	1,75598	-1,26384
Proteasome subunit beta type-4	PSMB4	3,1436	-0,890987
Mitogen-activated protein kinase 1	MAPK1	2,61753	-0,784326
Protein PML	PML	1,8994	0,901271
Elongation factor 1-delta	EEF1D	2,36834	1,27918
Endoplasmic reticulum resident protein 29	ERP29	2,92356	0,541735
High affinity immunoglobulin epsilon receptor subunit gamma	FCER1G	2,38926	0,746802
HLA class I histocompatibility antigen, B-53 alpha chain	HLA-B	2,7472	0,790903
Leukocyte elastase inhibitor	SERPINB1	3,1684	-0,859692
Coronin-1A	CORO1A	2,26719	0,63936
cAMP-dependent protein kinase type II-beta regulatory subunit	PRKAR2B	1,83878	0,900121
Cytochrome b-c1 complex subunit 1, mitochondrial	UQCRC1	3,39441	-0,660538
3-hydroxyisobutyrate dehydrogenase, mitochondrial	HIBADH	2,68811	-1,24953
14-3-3 protein beta/alpha	YWHAB	1,67599	1,40275
Cleavage stimulation factor subunit 2	CSTF2	1,74765	0,979084
Oxygen-dependent coproporphyrinogen-III oxidase, mitochondrial	CPOX	2,05769	-2,51356
DNA-directed RNA polymerase II subunit RPB9	POLR2I	2,39122	-0,987918
V-type proton ATPase catalytic subunit A	ATP6V1A	2,47061	-0,527253
T-complex protein 1 subunit zeta	CCT6A	2,77191	-0,578092
Malate dehydrogenase, mitochondrial	MDH2	3,06233	-0,384426
Trifunctional enzyme subunit alpha, mitochondrial	HADHA	3,86287	-0,949674
Tyrosine-protein kinase CSK	CSK	4,6583	-0,96348
Isoleucine--tRNA ligase, cytoplasmic	IARS	2,15091	-1,79023
Enoyl-CoA delta isomerase 1, mitochondrial	ECI1	2,26472	-1,16629
Leucine-rich PPR motif-containing protein, mitochondrial	LRPPRC	2,16936	-0,89247
3-ketoacyl-CoA thiolase, mitochondrial	ACAA2	3,14964	-0,626313

Glycerol-3-phosphate dehydrogenase, mitochondrial	GPD2	2,4452	-1,19893
Tyrosine-protein kinase SYK	SYK	3,76973	-0,901762
Chromobox protein homolog 5	CBX5	2,10899	1,08514
Probable 28S rRNA (cytosine(4447)-C(5))-methyltransferase	NOP2	1,6149	1,19726
Dual specificity mitogen-activated protein kinase kinase 3	MAP2K3	2,15019	-0,813216
Glucosamine-6-phosphate isomerase 1	GNPDA1	2,94327	-2,06763
F-actin-capping protein subunit alpha-2	CAPZA2	2,63881	-0,444223
Eukaryotic translation initiation factor 1A, X-chromosomal	EIF1AX	1,67984	1,34877
Glutamine--tRNA ligase	QARS	2,08611	-0,715545
Protein PRRC2A	PRRC2A	2,70659	0,880562
Signal recognition particle 9 kDa protein	SRP9	1,84039	0,937887
Alanine--tRNA ligase, cytoplasmic	AARS	2,04659	-0,991529
Serine--tRNA ligase, cytoplasmic	SARS	3,79332	-0,612337
Very long-chain specific acyl-CoA dehydrogenase, mitochondrial	ACADVL	2,39053	-0,604834
Selenide, water dikinase 1	SEPHS1	2,74704	-0,910801
Carnitine O-palmitoyltransferase 1, liver isoform	CPT1A	2,06977	-0,688274
Vasodilator-stimulated phosphoprotein	VASP	3,56988	0,618461
Dynamin-2	DNM2	2,5002	-0,707044
T-complex protein 1 subunit delta	CCT4	3,40388	-0,896176
Isocitrate dehydrogenase [NAD] subunit gamma, mitochondrial	IDH3G	1,7228	-1,38255
Host cell factor 1	HCFC1	2,33379	1,18256
Fatty aldehyde dehydrogenase	ALDH3A2	2,11063	-1,79095
Peroxisomal multifunctional enzyme type 2	HSD17B4	3,64602	-0,989813
Vesicle-associated membrane protein 7	VAMP7	1,57467	-1,24284
Ribosomal protein S6 kinase alpha-3	RPS6KA3	3,99285	-1,39619
Heterogeneous nuclear ribonucleoprotein M	HNRNPM	3,1392	0,613544
Heterogeneous nuclear ribonucleoprotein F	HNRNPF	2,54012	-0,572794
Hexokinase-3	HK3	2,82374	-0,598927
Tricarboxylate transport protein, mitochondrial	SLC25A1	2,76965	-1,40092
ATP-citrate synthase	ACLY	2,75442	-0,910218
Coatomer subunit beta	COPB1	1,98502	-0,845007

Coatomer subunit alpha	COPA	2,7146	-0,661044
Methylosome subunit pICln	CLNS1A	2,28733	1,07814
Tyrosine--tRNA ligase, cytoplasmic	YARS	2,22958	-0,564491
Adenosine kinase	ADK	2,61725	-2,0628
Puromycin-sensitive aminopeptidase	NPEPPS	2,30021	-0,695954
IgG receptor FcRn large subunit p51	FCGRT	1,48641	-1,68566
Methionine--tRNA ligase, cytoplasmic	MARS	1,7956	-1,01287
AP-1 complex subunit sigma-2	AP1S2	3,79375	-0,432019
Protein transport protein Sec61 subunit beta	SEC61B	2,96839	1,28473
Ribose-phosphate pyrophosphokinase 1	PRPS1	1,57901	-1,25981
Ubiquitin-conjugating enzyme E2 K	UBE2K	1,99856	-0,768679
Actin-related protein 3	ACTR3	2,80776	-0,519711
Alpha-centractin	ACTR1A	2,68743	-0,886678
COP9 signalosome complex subunit 2	COPS2	2,74075	-1,22798
Ras-related protein Rap-2b	RAP2B	1,65553	-1,45289
Protein max	MAX	3,66876	1,10512
60S ribosomal protein L27	RPL27	2,11708	-1,35084
40S ribosomal protein S7	RPS7	1,73713	-1,04498
Serine/threonine-protein phosphatase PP1-alpha catalytic subunit	PPP1CA	2,44289	-0,545941
40S ribosomal protein S13	RPS13	1,94349	-1,10148
Histone H4	HIST1H4A	2,47475	-0,484609
60S ribosomal protein L23	RPL23	3,64318	-1,41488
Guanine nucleotide-binding protein G(I)/G(S)/G(T) subunit beta-2	GNB2	2,74817	-0,609241
60S ribosomal protein L10a	RPL10A	2,34054	-0,890566
60S ribosomal protein L8	RPL8	2,59349	-0,765245
Peptidyl-prolyl cis-trans isomerase A	PPIA	2,46366	0,548413
Guanine nucleotide-binding protein G(s) subunit alpha isoforms short	GNAS	2,68883	-1,20628
Serine/threonine-protein phosphatase 2A 55 kDa regulatory subunit B alpha isoform	PPP2R2A	2,70699	-0,640564
40S ribosomal protein S21	RPS21	2,96434	0,700949
Guanine nucleotide-binding protein subunit beta-2-like 1	GNB2L1	2,4237	-1,0795
SUMO-conjugating enzyme UBC9	Ube2i	1,81859	-1,01179

Serine/threonine-protein phosphatase 2A catalytic subunit alpha isoform	PPP2CA	2,60837	-1,08207
Nuclease-sensitive element-binding protein 1	YBX1	3,09281	0,670355
Signal peptidase complex catalytic subunit SEC11A	SEC11A	2,20306	-1,17365
Tubulin beta-4B chain	TUBB4B	4,74287	-0,732298
Immunoglobulin-binding protein 1	IGBP1	1,80974	1,22574
Glutathione S-transferase omega-1	GSTO1	1,97652	-0,715778
DNA-dependent protein kinase catalytic subunit	PRKDC	3,60594	-0,776316
28S ribosomal protein S36, mitochondrial	MRPS36	3,33779	0,712056
Serine/arginine-rich splicing factor 3	SRSF3	1,921	0,936574
Histone H3.2	HIST2H3A	1,77461	-4,14398
Phosphate carrier protein, mitochondrial	SLC25A3	3,70505	-1,12527
Transcriptional activator protein Pur-alpha	PURA	2,49857	-0,721097
Methylmalonate-semialdehyde dehydrogenase [acylating], mitochondrial	ALDH6A1	1,81749	-0,926159
DNA topoisomerase 2-beta	TOP2B	2,03779	-1,06745
Single-stranded DNA-binding protein, mitochondrial	SSBP1	1,91343	-0,834792
Tyrosine-protein phosphatase non-receptor type 11	PTPN11	2,18192	-0,681671
Cytoskeleton-associated protein 4	CKAP4	2,28852	0,70689
Mitochondrial-processing peptidase subunit alpha	PMPCA	1,98611	-1,26331
Vesicular integral-membrane protein VIP36	LMAN2	2,84059	-1,80768
Heat shock protein 75 kDa, mitochondrial	TRAP1	2,20098	-1,40645
Protein flightless-1 homolog	FLII	2,88011	-0,990279
Lymphocyte cytosolic protein 2	LCP2	2,46652	0,61867
Ubiquitin carboxyl-terminal hydrolase 4	USP4	2,06133	-1,2616
Serine/arginine-rich splicing factor 9	SRSF9	2,43055	0,998055
Serine/arginine-rich splicing factor 6	SRSF6	2,05764	0,961327
Voltage-gated potassium channel subunit beta-2	KCNAB2	1,86417	-1,03915
Cytoplasmic dynein 1 intermediate chain 2	DYNC1I2	2,2055	0,565186
V-type proton ATPase 116 kDa subunit a isoform 3	TCIRG1	2,73568	-1,00609
Diacylglycerol kinase zeta	DGKZ	2,18066	-1,16102
Stromal interaction molecule 1	STIM1	2,51708	0,529726
Bleomycin hydrolase	BLMH	2,4213	-2,6743

Ubiquitin-associated protein 2-like	UBAP2L	2,51005	0,794757
Cytoplasmic dynein 1 heavy chain 1	DYNC1H1	2,08893	-0,718312
Guanine nucleotide-binding protein subunit alpha-13	GNA13	3,499	-1,02924
RNA-binding protein 39	RBM39	2,35072	-0,576593
Protein disulfide-isomerase A5	PDIA5	2,29842	-1,65076
Pre-mRNA-splicing regulator WTAP	WTAP	3,47656	1,00766
Septin-2	02-set	2,73964	-0,493138
116 kDa U5 small nuclear ribonucleoprotein component	EFTUD2	2,0319	-0,905758
Neutrophil cytosol factor 4	NCF4	2,8733	-0,884125
Transcription elongation factor B polypeptide 2	TCEB2	2,40538	0,768398
Ubiquitin-protein ligase E3C	UBE3C	1,86271	-0,88677
Scaffold attachment factor B1	SAFB	2,39901	0,49421
Splicing factor 1	SF1	2,1738	0,615689
ELAV-like protein 1	ELAVL1	1,98018	-0,963947
NEDD8	NEDD8	2,17462	0,993753
39S ribosomal protein L23, mitochondrial	MRPL23	2,08323	-1,19942
Hsp90 co-chaperone Cdc37	CDC37	2,66815	0,4987
Kynureninase	KYNU	2,52648	-1,09881
NADH dehydrogenase [ubiquinone] 1 alpha subcomplex subunit 9, mitochondrial	NDUFA9	3,07419	-1,64304
UTP--glucose-1-phosphate uridylyltransferase	UGP2	3,22477	-1,51934
Macrophage-expressed gene 1 protein	MPEG1	1,79612	-0,976171
Protein LSM12 homolog	LSM12	1,8273	2,12645
Putative oxidoreductase GLYR1	GLYR1	2,53032	-0,515744
Presequence protease, mitochondrial	PITRM1	2,03165	-1,9872
RRP12-like protein	RRP12	1,46907	-1,74184
Engulfment and cell motility protein 2	ELMO2	2,17375	-0,731397
FAD synthase	FLAD1	1,5441	-1,37372
Microtubule-associated protein 1S	MAP1S	1,50047	-2,20191
Atlastin-3	ATL3	2,73621	-1,72114
Twinfilin-2	TWF2	3,72044	-0,856113
Pre-mRNA-processing-splicing factor 8	PRPF8	2,36247	-0,940774

La-related protein 1	LARP1	2,51979	1,01923
Biogenesis of lysosome-related organelles complex 1 subunit 2	BLOC1S2	1,70029	1,06084
Nesprin-3	SYNE3	2,01681	-0,676152
Neurobeachin-like protein 2	NBEAL2	1,9098	-1,54626
Vacuolar protein sorting-associated protein 13C	VPS13C	2,82177	-1,00394
Protein unc-13 homolog D	UNC13D	2,62309	-0,666353
Staphylococcal nuclease domain-containing protein 1	SND1	3,56017	-0,578772
Eukaryotic translation initiation factor 3 subunit M	EIF3M	1,65273	1,27447
Zinc finger CCCH-type antiviral protein 1	ZC3HAV1	2,8254	0,726645
Elongation factor Tu GTP-binding domain-containing protein 1	EFTUD1	2,20326	-1,65264
Myosin-14	MYH14	1,99775	-1,29583
KDEL motif-containing protein 2	KDELC2	1,85801	-0,96589
Protein LYRIC	MTDH	2,63261	0,528735
Cullin-associated NEDD8-dissociated protein 1	CAND1	1,98656	-1,02126
Ankyrin repeat and KH domain-containing protein 1	ANKHD1	3,26598	1,26275
Chromatin complexes subunit BAP18	BAP18	3,35271	0,550715
EH domain-binding protein 1-like protein 1	EHBP1L1	3,50919	0,47355
Cohesin subunit SA-2	STAG2	2,061	-1,67936
ADP-ribosylation factor GTPase-activating protein 1	ARFGAP1	2,31082	0,69476
Serine/threonine-protein phosphatase 6 regulatory ankyrin repeat subunit B	ANKRD44	1,72405	-1,3506
Parkinson disease 7 domain-containing protein 1	PDDC1	1,74804	-1,05617
NHL repeat-containing protein 2	NHLRC2	1,87036	-0,990679
Saccharopine dehydrogenase-like oxidoreductase	SCCPDH	3,26675	-1,76188
Dedicator of cytokinesis protein 8	DOCK8	2,96461	-0,979904
Nucleoporin NUP53	NUP35	2,30424	0,672071
E3 ubiquitin-protein ligase ZNRF2	ZNRF2	2,23478	1,35541
Splicing factor U2AF 26 kDa subunit	U2AF1L4	1,9353	1,16037
Nuclear pore complex protein Nup133	NUP133	1,67874	-1,50911
Sec1 family domain-containing protein 1	SCFD1	2,1278	-1,41071
PEST proteolytic signal-containing nuclear protein	PCNP	2,10385	0,909601
GTPase IMAP family member 1	GIMAP1	1,6353	-1,29585

UBX domain-containing protein 4	UBXN4	2,28551	0,822291
Dedicator of cytokinesis protein 2	DOCK2	2,89095	-0,907926
Translational activator GCN1	GCN1L1	5,08812	-0,941544
Pre-mRNA-splicing factor ATP-dependent RNA helicase PRP16	DHX38	2,34844	-1,71038
Ubiquitin carboxyl-terminal hydrolase 7	USP7	2,19789	-2,06639
Transcription elongation factor A protein-like 3	TCEAL3	1,95649	1,21938
Synapse-associated protein 1	SYAP1	1,93329	0,731824
Vacuolar protein sorting-associated protein 33A	VPS33A	2,24503	-2,17975
U8 snoRNA-decapping enzyme	NUDT16	3,72204	-2,43102
Copine-2	CPNE2	2,28787	-1,11777
RNA-binding protein 14	RBM14	2,82584	0,479664
Vacuolar protein sorting-associated protein 35	VPS35	1,98205	-0,870391
GPI transamidase component PIG-S	PIGS	2,46451	-1,59008
Chloride channel CLIC-like protein 1	CLCC1	1,8732	0,925029
3-hydroxyacyl-CoA dehydrogenase type-2	HSD17B10	3,18232	-1,11657
Histone H2B type 1-M	HIST1H2BM	1,6272	3,96336
Tyrosine-protein phosphatase non-receptor type 18	PTPN18	2,54493	1,35423
Protein NipSnap homolog 1	NIPSNAP1	2,32923	-1,65041
Serine/threonine-protein phosphatase CPPED1	CPPED1	2,09883	-0,678389
Partner of Y14 and mago	WIBG	2,88337	0,534585
ADP-dependent glucokinase	ADPGK	3,18701	-1,66906
COP9 signalosome complex subunit 4	COPS4	2,42094	-0,875684
Mini-chromosome maintenance complex-binding protein	MCMBP	1,96243	0,679536
Transmembrane protein 43	TMEM43	1,66103	-1,50206
Charged multivesicular body protein 4a	CHMP4A	2,67597	0,6476
RanBP-type and C3HC4-type zinc finger-containing protein 1	RBCK1	1,6776	1,56482
E3 ubiquitin-protein ligase TRIM4	TRIM4	1,64549	-1,45091
Phospholysine phosphohistidine inorganic pyrophosphate phosphatase	LHPP	1,72819	-1,87908
Thioredoxin-related transmembrane protein 4	TMX4	2,10657	-1,46578
Vacuolar protein sorting-associated protein 16 homolog	VPS16	2,68981	-1,46442
Tubulin beta-1 chain	TUBB1	1,84806	-2,94456

39S ribosomal protein L44, mitochondrial	MRPL44	2,38241	-0,816819
Nicotinamide/nicotinic acid mononucleotide adenylyltransferase 1	NMNAT1	2,1957	-1,5928
Calcyclin-binding protein	CACYBP	1,84313	1,10445
Manganese-transporting ATPase 13A1	ATP13A1	2,7988	-0,725937
Charged multivesicular body protein 1a	CHMP1A	2,1657	3,30591
Toll-like receptor 8	TLR8	1,93982	-1,10091
F-box only protein 6	FBXO6	1,68245	2,35402
Epimerase family protein SDR39U1	SDR39U1	1,86874	-1,4544
Protein FAM114A2	FAM114A2	2,28072	-0,888541
Phenylalanine--tRNA ligase beta subunit	FARSB	2,17824	-0,909596
Ethylmalonyl-CoA decarboxylase	ECHDC1	5,48376	-1,26085
Mycophenolic acid acyl-glucuronide esterase, mitochondrial	ABHD10	1,72156	-1,58812
ADP-ribosylation factor-like protein 8B	ARL8B	1,74521	-0,919146
ATP-dependent RNA helicase DDX18	DDX18	1,75179	-1,34623
THUMP domain-containing protein 1	THUMPD1	2,116	0,930059
Nuclear distribution protein nudE homolog 1	NDE1	2,31384	-0,925975
UDP-glucose:glycoprotein glucosyltransferase 1	UGGT1	2,71331	-0,868593
Adenosine deaminase CECR1	CECR1	2,8293	-1,63256
E3 ubiquitin-protein ligase RNF181	RNF181	2,96526	1,61198
Vacuolar protein sorting-associated protein 18 homolog	VPS18	1,74559	0,875457
Leucine--tRNA ligase, cytoplasmic	LARS	3,54143	-2,0452
Vacuolar protein sorting-associated protein 29	VPS29	1,98758	-1,37989
Cathepsin Z	CTSZ	1,90528	0,921716
Protein NipSnap homolog 3A	NIPSNAP3A	2,33258	-2,12625
Signal recognition particle subunit SRP68	SRP68	1,75838	-0,944346
Vacuolar protein sorting-associated protein 51 homolog	VPS51	1,81778	-2,21265
Tyrosine-protein kinase BAZ1B	BAZ1B	1,60595	-1,14092
Hematological and neurological expressed 1 protein	HN1	3,45913	0,744209
Apoptosis-associated speck-like protein containing a CARD	PYCARD	1,53768	-1,35594
26S proteasome non-ATPase regulatory subunit 13	PSMD13	2,83225	-1,62535
Zinc finger CCCH domain-containing protein 4	ZC3H4	3,04223	0,711782

Translation machinery-associated protein 7	TMA7	2,82494	1,09774
AP-3 complex subunit mu-1	AP3M1	2,04928	-0,973914
Thyroid hormone receptor-associated protein 3	THRAP3	2,15996	0,623545
Acyl-coenzyme A thioesterase 9, mitochondrial	ACOT9	2,79518	-0,808127
Deoxyribose-phosphate aldolase	DERA	1,83758	-1,00717
Mitochondrial fission 1 protein	FIS1	2,46892	-1,03182
Deoxynucleoside triphosphate triphosphohydrolase SAMHD1	SAMHD1	2,76034	-0,5844
Unconventional myosin-Va	MYO5A	2,22747	-1,72203
Leucine-rich repeat flightless-interacting protein 2	LRRFIP2	2,55254	1,61824
Mitochondrial carrier homolog 2	MTCH2	2,68093	-1,74277
Sulfide:quinone oxidoreductase, mitochondrial	SQRDL	5,68902	-0,769156
Apoptotic chromatin condensation inducer in the nucleus	ACIN1	2,72152	0,584889

Table S7								
**Genes related to FMF**	**UNT**	**LPS**	**Interesting genes**	**UNT**	**LPS**	**Others**	**UNT**	**LPS**
MEFV			IFIT1			MRPL17		
PYCARD			IFIT2			RPL9		
IL1RN			IFIT3			RPL35A		
IL1B			ISG15			RPL36		
CASP1			MB21D1			RPS15		
IL18			PRDX6			PPIE		
TNF			GNAQ			YY1		
PSTPIP1			SHC1			DAXX		
RHOA			PTPN11			HAT1		
IKBKB			MIF			HIST2H3A		
MAPK3			CDK9			HIST1H2BK		
STAT3			FN1			RB1		
STAT5B			ELANE			SHDB		
PIK3R1			PPP1CA			GUSB		
PKN1			SHARPIN			LYZ		
PRKCA			RHOT1			Ca2		
TLR2			TMEM173			TUBB2A		
S100A12			TAX1BP3			SPN		
C5AR1			CUL3			CCNY		
DNM1L						ERAP1		
TNFRSF1B						TTC37		
TTR						GRHPR		
TPT1						ELL		
TRIM21								
B2M								
SLC25A1								

Table S8								
**Genes related to TRAPS**	**UNT**	**LPS**	**Interesting genes**	**UNT**	**LPS**	**Others**	**UNT**	**LPS**
MEFV			TTR			SF3A1		
PYCARD			CRK			SF3B5		
TNFRSF1B			YWHAQ			SRSF4		
ADAM17			OPTN			SRSF11		
STAT3			VAV1			SRSF6		
TPT1			RAP1A			NUP43		
LYZ			USP9X			RPS15		
IKBKB			ACTR1A			LYPLA2		
			SRP9			RPL35A		
			EIF3M			RPLP2		
			LYN			UFC1		
						C14orf166		

Table S9

Genes related to MVD	UNT	LPS	Interesting genes	UNT	LPS	Others	UNT	LPS
IL1RN			LYN			RPL8		
IL1B			SYK			CLNS1A		
PSTPIP1			GNA13			MRPL44		
MAPK1			GNA12			HIST2H3A		
MAP2K3			PTPN18			PIGS		
CD14			PTPN11			SEPHS1		
NFKB1			VAV1			QARS		
RHOA			PSMB4			TARS		
LRPAP1			ACTR1A			YARS		
TMEM173			ACTR3A			HSD17B10		
TLR2			VASP			HSPD1		
TLR8			SEC61B			LMNB1		
RAB14			XRCC5			FLOT1		
RAB7			SAMHD1			PGRMC1		
RAB1A			NEDD8			AP1G2		
ATG7			USP4			DCTN6		
Mefv			RBCK1			MFN2		
Pycard			TCEB2			MTCH2		
TNFRSF1B			ZNRF2			HNRNPF		
Casp1			UBE3C			CTSD		
IL18			TRIM4			SRSF3		
TNF			LMAN2			CPNE2		
			RAP2B			MTDH		
			NCF4			CA2		
			SND1			MYO1F		
			FBXO6			MYO1G		
			CPT1A			MYO5A		
			CPT2			LRPPRC		
			ECI1			TCP1		
			CSK			TOP2B		
			DNM2			NCL		
			VPS16			NME2		
			VPS33A			ELAVL1		
			VPS29			UQCRC2		
			VPS18			NDUFA9		
			STX7			NDUFS2		
			ATP6V1A			CBX5		
			CTSZ			SLC25A3		
			XPO1			HMGA1		
			ARL8B			LSM12		
			CAPZA2			MMAB		
			VAMP7			MSN		

			AP3M1			IDH3G		
			COPA			ACADVL		
			COPB1			ECHDC1		
			WDR1			IMPDH2		
			CAPN1			MT-CO2		
			SEC23B			ALDH2		
			AP2A1			ALDH3A2		
			TUBB1			ALDH6A1		
			TUBB4B					
			DYNC1H1					
			RPN2					
			SLC25A1					
			RPS6KA3					
			YWHAB					
			PRKAR2B					
			ACLY					
			HADHA					
			VDAC1					
			LBR					
			FDPS					
			HSD17B4					
			ACAA2					
			FAH					
			ITGAL					
